# Circadian regulation of key physiological processes by the RITMO1 clock protein in the marine diatom *Phaeodactylum tricornutum*

**DOI:** 10.1111/nph.70099

**Published:** 2025-04-02

**Authors:** Alessandro Manzotti, Raphaël Monteil, Soizic Cheminant Navarro, Dany Croteau, Lucie Charreton, Antoine Hoguin, Nils Fabian Strumpen, Denis Jallet, Fayza Daboussi, Peter G. Kroth, François‐Yves Bouget, Marianne Jaubert, Benjamin Bailleul, Jean‐Pierre Bouly, Angela Falciatore

**Affiliations:** ^1^ Laboratoire de Photobiologie et Physiologie des Plastes et des Microalgue, UMR7141 CNRS, Sorbonne Université, Institut de Biologie Physico‐Chimique 75005 Paris France; ^2^ Fachbereich Biologie Universität Konstanz Konstanz 78457 Germany; ^3^ Toulouse Biotechnology Institute (TBI) Université de Toulouse, CNRS, INRAE, INSA 31077 Toulouse France; ^4^ Toulouse White Biotechnology (TWB), INSA 31077 Toulouse France; ^5^ Laboratoire d'Océanographie Microbienne Sorbonne Université, CNRS, UMR7621, Observatoire Océanologique 66650 Banyuls sur Mer France; ^6^ Molécules de Communication et Adaptation des Micro‐Organismes, UMR 7245, CNRS/MNHN F‐75231 Paris France

**Keywords:** biological rhythms, circadian clock, diatom, gene expression, photophysiology, photosynthesis, phytoplankton, RITMO1

## Abstract

- Phasing biological and physiological processes to periodic light–dark cycles is crucial for the life of most organisms. Marine diatoms, as many phytoplanktonic species, exhibit biological rhythms, yet their molecular timekeepers remain largely uncharacterized. Recently, the bHLH‐PAS protein RITMO1 has been proposed to act as a regulator of diatom circadian rhythms.
- In this study, we first determined the physiological conditions to monitor circadian clock activity and its perturbation in the diatom model species *Phaeodactylum tricornutum* by using cell fluorescence as a circadian output. Employing ectopic overexpression, targeted gene mutagenesis, and functional complementation, we then investigated the role of RITMO1 in various circadian processes.
- Our data reveal that RITMO1 significantly influences the *P. tricornutum* circadian rhythms not only of cellular fluorescence, but also of photosynthesis and of the expression of clock‐controlled genes, including transcription factors and putative clock input/output components. RITMO1 effects on rhythmicity are unambiguously detectable under free‐running conditions.
- By uncovering the complex regulation of biological rhythms in *P. tricornutum,* these findings advance our understanding of the endogenous factors controlling diatom physiological responses to environmental changes. They also offer initial insights into the mechanistic principles of oscillator functions in a major group of phytoplankton, which remain largely unexplored in chronobiology.

## Introduction

Diatoms are unicellular algae that can colonize all aquatic environments, while also being found in sea ice, mudflats and soils (Pierella Karlusich *et al*., 2020). With an estimated number of > 100 000 species (Malviya *et al*., 2016), diatoms represent the most species‐rich algae, playing a central role in trophic networks and biogeochemical cycles of the oceans. Although being predominantly photosynthetic, diatoms are not directly related to terrestrial plants and green algae of the Archaeplastida clade, which are derived by a primary endosymbiosis event. Like other Stramenopiles, diatoms acquired their plastid through endosymbiotic events involving a eukaryotic red and possibly a green alga taken up by a heterotrophic host cell (Moustafa *et al*., 2009; Burki *et al*., 2020). Traces of this complex evolutionary history are found in their peculiar gene repertoire derived from their heterotrophic and phototrophic ancestors, as well as from bacteria via horizontal gene transfer (Bowler *et al*., 2008; Mock *et al*., 2022).

Circadian clocks are endogenous timing systems that generate biological cycles of *c*. 24 h, identified in a wide variety of organisms, from bacteria to humans (Dunlap, 1999; Bell‐Pedersen *et al*., 2005). These clocks involve many different mechanisms and molecular players, yet they usually share common features. In eukaryotes, they are mainly structured around interlocked negative transcriptional‐translational feed‐back loops (TTFL) that use transcription factors (TFs) to control the expression of cognate genes (Dunlap, 1999). Besides, non‐TTFL‐based oscillatory systems also exist (Sweeney & Haxo, 1961; Ishiura *et al*., 1998; Edgar *et al*., 2012). Periodic light and dark cycles represent the primary time giver (‘Zeitgeber time’, ZT), with light‐sensing receptors acting in the input pathways to synchronize the circadian clock to diel light rhythms (Millar *et al*., 1995; Oakenfull & Davis, 2017). In addition to light, other abiotic factors, such as temperature, can play an influential role in clock entrainment (Rensing & Ruoff, 2002). The clock regulatory loop is self‐sustained, that is oscillations of the endogenous clock, as well as controlled physiological processes, are maintained for several days after exposure to a constant environment with no light : dark (LD) or temperature cycles. In photosynthetic organisms, the circadian clock system has been extensively characterized in the model plant species *Arabidopsis thaliana*, where intricated regulatory feedback loops control the rhythmicity of major metabolic and cellular processes (Dodd *et al*., 2005; Fung‐Uceda *et al*., 2018; de Barros Dantas *et al*., 2023), as well as the developmental transition to flowering (Song *et al*., 2015). In algae, timekeeper components have so far been elucidated in detail only in representative model species of the green lineage derived by primary endosymbiosis such as the Chlorophyceae *Chlamydomonas reinhardtii* and the Mamiellophyceae *Ostreococcus tauri* (Petersen *et al*., 2022). The latter time keeping mechanism relies on a minimalist plant‐like clock with only two components, OtCCA1 and OtTOC1 (Corellou *et al*., 2009).

There is compelling evidence that diatoms, like other phototrophs, have evolved to adjust their physiology and metabolism to periodic LD fluctuations. Under laboratory conditions, diel rhythms have been described in diverse species for essential processes such as photosynthesis (Post *et al*., 1984; Jallet *et al*., 2016), pigment synthesis (Owens *et al*., 1980; Hunsperger *et al*., 2016), cell division (Nelson & Brand, 1979), and gene expression (Chauton *et al*., 2013; Smith *et al*., 2016; Bilcke *et al*., 2021). *In situ* studies of natural populations have also reported rhythms in optical properties related to diatom photosynthetic activity, growth (Brand, 1982; Kheireddine & Antoine, 2014), and gene expression, revealed by diel metatranscriptomics analyses (Kolody *et al*., 2019; Coesel *et al*., 2021). Nevertheless, the involvement of endogenous timekeeper(s) in the regulation of these rhythms has remained unclear until recently. This uncertainty was partly due to the very limited data on rhythm persistence under free‐running conditions (Palmer *et al*., 1964; Nelson & Brand, 1979; Chisholm & Brand, 1981; Ragni & d'Alcalà, 2007; Häfker *et al*., 2023), a key feature of the clock function, and the lack of effective automated methods to study circadian processes. Furthermore, with the exception of *Cryptochromes* and *Casein Kinases* (Coesel *et al*., 2009; Farré, 2020; Jaubert *et al*., 2022), diatom genomes lack key clock genes found in other systems. However, gene expression analyses of target genes in the model species *Phaeodactylum tricornutum* under constant darkness (Annunziata *et al*., 2019; Madhuri *et al*., 2024), alongside transcriptomic analyses under constant light illumination (Su *et al*., 2024), have recently pointed to transcriptional regulation of diatom circadian rhythms. It has been proposed that, despite the high divergence, certain diatom proteins containing domains typically found in eukaryotic clock components may have acquired functions related to circadian clock regulation. One such example is the RITMO1 protein (Annunziata *et al*., 2019), which, similarly to central animal clock proteins (Takahashi, 2017), possesses bHLH‐PAS domains. RITMO1 was first identified in a survey of the most rhythmic TFs in *P. tricornutum* (Annunziata *et al*., 2019). Since, multiple *RITMO1*‐like bHLH‐PAS genes have been found in all diatoms and other Stramenopiles for which omic sequences are available, as well as in other phytoplanktonic organisms of different phyla (Annunziata *et al*., 2019; Farré, 2020; Bilcke *et al*., 2021). Deregulation of *RITMO1* expression levels and timing resulted in *P. tricornutum* cells with altered gene expression profiles in LD cycles but also in continuous darkness (DD), showing that the effect of RITMO1 on rhythmicity is not directly dependent on light inputs (Annunziata *et al*., 2019). Initial analyses of cellular fluorescence rhythms, which in diatoms reflect synchrony in plastid ontogeny during cell cycle (Ragni & d'Alcalà, 2007; Huysman *et al*., 2013; Hunsperger *et al*., 2016), also revealed that RITMO1 influences circadian rhythms (Annunziata *et al*., 2019), suggesting a possible function for this protein in the circadian clock. Exclusively found in Stramenopiles (Takahashi *et al*., 2007; Ishikawa *et al*., 2009), the Aureochrome1a (AUREO1a) photoreceptor, which contains both a blue light‐sensing LOV domain and a DNA‐binding bZIP domain, has also been proposed to be involved in diatoms circadian regulation (Madhuri *et al*., 2024). While AUREO1a is known for its central role in the *P. tricornutum* blue light responses (Kroth *et al*., 2017; Mann *et al*., 2020), PtAUREO1a knock‐out lines have also been found to exhibit perturbed rhythmic expression of several genes, including *RITMO1* and Cryptochrome Photolyase *CPF1* in both LD and DD (Madhuri *et al*., 2024).

In this study, we further characterize the circadian clock function in diatoms. Alongside *RITMO1* over‐expressing lines, we generated RITMO1 loss of function mutants in *P. tricornutum* using CRISPR/Cas9 editing. We then tested the consequence of these modifications for various biological rhythms under different LD cycles and free‐running conditions. Further supported by complementation tests in *RITMO1* mutants, our findings revealed a tight molecular control of rhythmic events in *P. tricornutum* and a key role for RITMO1 in the circadian regulation of diatom physiology.

## Materials and Methods

### Cultures conditions

Transgenic and wild‐type (WT) lines of *Phaeodactylum tricornutum,* Bohlin, used in this study refer to strain Pt1 8.6; CCMP2561 (Bowler *et al*., 2008), with the exception of the *PtAUREO1a* knock‐out mutants generated in the UTEX646 Pt4 strain by Madhuri *et al*. (2024). Cells were cultured in F/2 media (Guillard, 1975) at 18°C under white light (Bridgelux BXRV‐DR‐1830H‐3000‐A‐13 LEDs) of different intensities and photoperiods, as detailed in the text. Cells were preadapted at least 2 wk to specific conditions before performing analyses of rhythmicity. For most of the experiments, batch cultures were grown in incubators with orbital agitation and analyses performed in cells in exponential phase of growth (between 0.1 and 2 × 10^6^ cell ml^−1^). Temperature compensation experiments were performed on cells entrained under 16 h : 8 h, LD cycles, 25 μmol photons m^−2^ s^−1^ at 18°C and subsequently exposed to LL of 17 μmol photons m^−2^ s^−1^ at different temperature ranges (14–22°C). Low temperature shift was done at the last LD transition (ZT 16) and at the higher temperature at the last DL transition (ZT 24) (Poliner *et al*., 2019). Entrainment experiments by both temperature and light cues were performed on cells adapted to 16 h : 8 h, LD, 25 μmol photons m^−2^ s^−1^, where the L phase temperature was set at 18°C and the D phase at 14°C. Following entrainment, cells were transferred to LL at 17 μmol photons m^−2^ s^−1^ and 18°C. For studies of photosynthesis, cells were grown in turbidostat modules (Multi‐Cultivator MC 1000‐OD; Photon Systems Instruments, Drasov, Czech Republic) and maintained at an optical density of 1–2 × 10^6^ cell ml^−1^. For all the experiments, cell concentrations were measured using a Z2 Coulter Particle Count and Size Analyser (Beckman Coulter, Brea, CA, USA). Growth capacity of the different strains was calculated during the exponential phase of growth over 4 d and expressed as number of divisions per day: log_2_ (*C*_4_/*C*_0_)/4, where *C*_0_ and *C*_4_ represent the culture concentrations at Days 0 and 4, respectively.

### Generation of RITMO1 mutant strains

RITMO1 mutants were generated by biolistic delivery of the Clustered Regularly Interspaced Short Palindromic Repeats (CRISPR)/Cas9 ribonucleoproteins according to Serif *et al*. (2018). Selection of transformed lines was accomplished by co‐targeting the *APT* gene (Phatr3_J6834) to induce resistance to 2‐fluoroadenine (2‐FA). Three and two independent sgRNAs were co‐transformed to target *RITMO1* and *APT*, respectively (Supporting Information Table S1). Eight 2‐FA resistant colonies were sequenced and four of them appeared to bear mutations on the two alleles of *RITMO1*. Wild‐type *P. tricornutum* cells, the KO1 and KO2 lines, an additional transgenic line showing no mutation in *RITMO1* (chosen as control, CTR line) have been characterized in this study. Moreover, rhythms have been also studied in a previously generated *RITMO1* ectopic overexpression line (OE1) (Annunziata *et al*., 2019). A RITMO1 complemented line (named KO2‐C2) was obtained by transforming KO2 with a construct containing the genomic *RITMO1* sequence fused to the *Venus* reporter, under the endogenous *RITMO1* promoter and terminator and characterized as described in Methods S1.

### Cellular fluorescence analysis

In circadian experiments, culture samples were automatically collected every 2 h via a BVP standard ISM444 peristaltic pump (Ismatec, Glattbrugg, Switzerland) controlled by a FC204 fraction collector (Gilson, Middleton, WI, USA). Before cytometry analysis, cells were stored at 4°C in the dark for at least 2 h to a maximum of 24 h. Cellular fluorescence rhythmicity (FL3 parameter) analyses were performed using a MACSQuant flow cytometer (Miltenyi Biotec, Bergisch Gladbach, Germany) (Annunziata *et al*., 2019). FL3‐A parameter (488 nm excitation, 655–730 nm detection) was used to track Chl fluorescence. Between 10 000 and 30 000 events were counted at each time point and for each culture replicate and median FL3‐A value were used for following rhythmicity.

### Analysis of photosynthetic parameters

For photophysiology characterisations, cells were grown in turbidostat modules, adapted to 16 h : 8 h, LD (50 μmol photons m^−2^ s^−1^) 4 d before the beginning of sampling and then shifted to continuous light (30 μmol photons m^−2^ s^−1^). Analyses were performed on four replicas of each genotype, measured every 15 min, always in the order WT, KO1, KO2, and OE1. Therefore, a given strain was sampled every 4 h but each genotype was sampled every hour. The samples remained for 1 min in darkness for relaxation before the measurements in the Fluorescence Induction and Relaxation fluorometer (miniFIRe; Gorbunov *et al*., 2020). A saturating single turnover flash (100 μs) of blue light (450 nm) applied on dark‐acclimated samples induced a fluorescence induction from *F*_0_ (extrapolated minimum fluorescence) to *F*_m_ (maximum fluorescence), which is then used to calculate the maximum quantum yield as *F*_v_/*F*_m_ = (*F*_m_ – *F*_0_)/*F*_m_. Then, a rapid light curve protocol was performed consisting of nine steps of increasing light intensities (*E*) (from 25 to 800 μmol photons m^−2^ s^−1^) with 1 min exposure duration for the first two light steps, followed by 20 s for the 7 next steps. Different photosynthetic parameters were calculated, including the light irradiance at half saturation of the non‐photochemical quenching (NPQ) (E50NPQ) and the NPQ relaxation after 4 min in the dark (NPQ rel.) as described in Methods S2 and Fig. S1.

### Analysis of rhythmic processes

The analysis of FL3‐A fluorescence rhythmicity was performed using the BioDare2 webtool (https://biodare2.ed.ac.uk/, Zielinski *et al*., 2014). Data traces were first analyzed using the Enright Periodogram (EPR) algorithm, which was used as an arrhythmicity test (Zielinski *et al*., 2014). Tracks not identified as rhythmic by EPR were used for graphical representations of average fluorescence, but were not further analyzed for period, amplitude and relative amplitude errors (RAE). Period, amplitude and RAE were calculated via fast Fourier transform non‐linear least square algorithm (FFT‐NLLS), within a period range of 18–34 h. At least three full oscillations were considered for the analysis. RAE, defined as the ratio of the amplitude error to the most probable derived amplitude magnitude (Plautz *et al*., 1997), was used as a measure of degree of confidence in the fit used by the algorithm to calculate rhythmic parameters. Statistical differences were examined using unpaired Student's *t*‐test with the WT as reference sample. For graphical representations, data traces were baseline detrended, normalized to the maximum value and aligned to the mean values.

For the analysis of photosynthesis, due to the constraints of the manual sampling, a limited number of cycles have been analyzed, preventing the appropriate use of the BioDare2 tool. Thanks to the high temporal sampling resolution, however, we were able to conduct rhythmicity tests using the JTK_CYCLE algorithm (v.3.1) (Hughes *et al*., 2010), by using the ‘replica option’. For the period in 16 h : 8 h, LD conditions, a period of 24 h was enforced. In LL, because circadian clock free‐runs with its own period, which does not match exactly 24 h, the period windows was set between 20 and 28 h.

### RNA extraction and RT‐qPCR analysis

For RNA purification, 10^8^ cells from exponential phase cultures were filtered onto Whatman 589/2 filters. RNA was extracted with TriPure Isolation Reagent (Roche) following the provided protocol and further purified by an ammonium acetate precipitation. Five hundred nanograms of RNA were reverse‐transcribed using an oligo‐dT and random primers mix and the QuantiTect Reverse Transcription Kit (Qiagen). For RT‐qPCR, the 30S ribosomal small subunit protein (*RPS*) and TATA box Binding Protein (*TBP*) were used as reference genes (Siaut *et al*., 2007). For each sample, the geometrical average of *C*_t_ was used as reference to evaluate the expression of genes of interest via the ∆∆*C*_t_ Livak method (Livak & Schmittgen, 2001). For each gene, data was normalized against the maximum expression value among the different strains, so that expression values are all between 0 and 1. This normalisation method allows to compare the relative gene expression among different cell lines. RT‐qPCR analyses were performed on two biological replicates in LD samples, and on three replica for experiments in LL and with the complemented line. Primers for RT‐qPCR analysis are listed in Table S1.

## Results

### Unveiling the experimental conditions for characterising the circadian clock in *P. tricornutum*

**Fig. 1:**
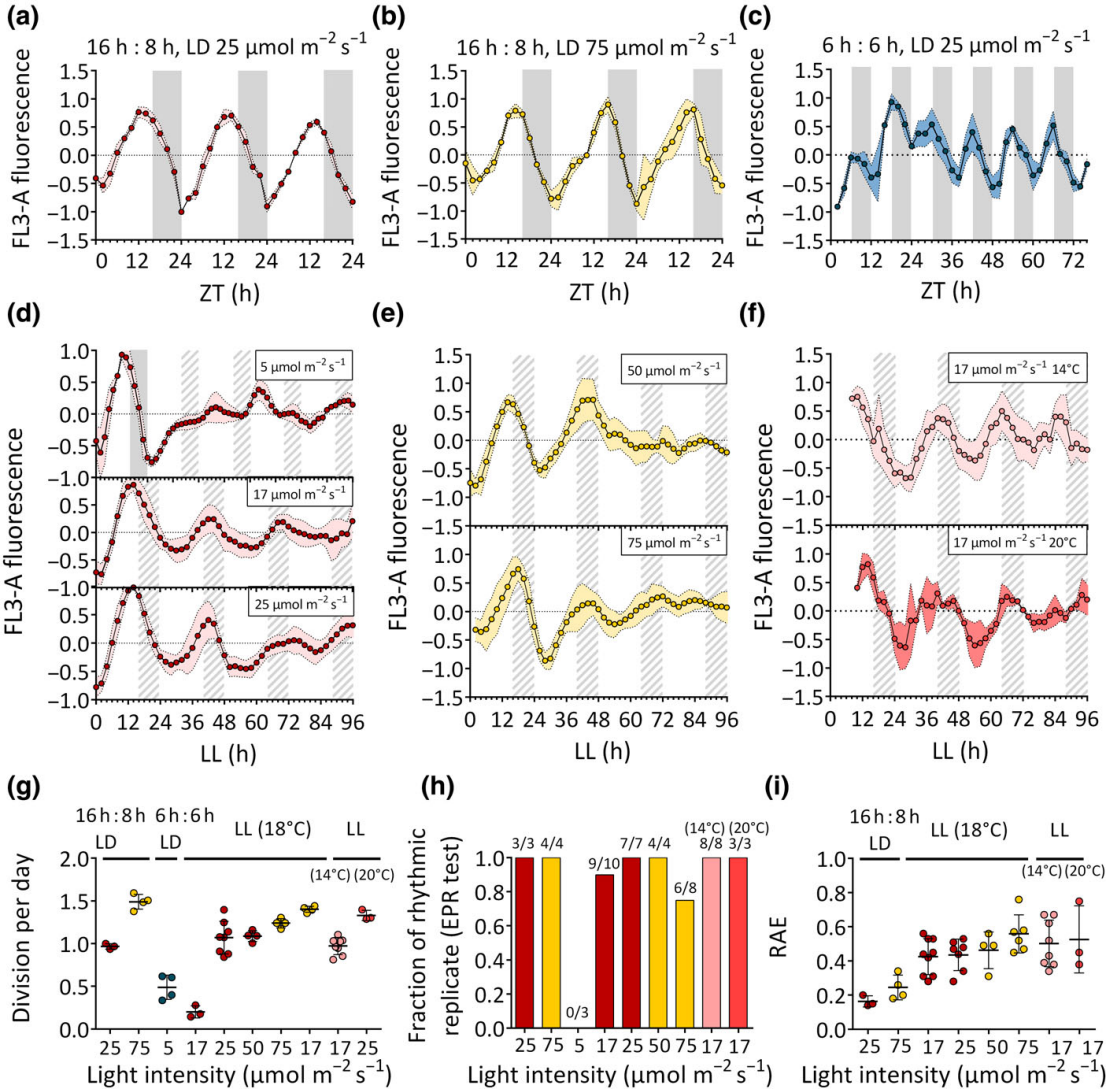
Characterisation of cellular fluorescence rhythmicity of *Phaeodactylum tricornutum* grown under different light : dark (LD) cycles of different light intensities and periods and following a shift to different free‐running conditions. (a) Fluorescence rhythms (FL3‐A) of cells entrained in 16 h : 8h, LD cycles at 25 μmol photons m^−2^ s^−1^ 18°C and (b) at 75 μmol photons m^−2^ s^−1^ 18°C; In (c) FL3‐A trends of cells shifted to 6 h : 6 h, LD cycles at 25 μmol photons m^−2^ s^−1^ 18°C after prior entrainment under 12 h : 12 h, LD cycles at the same intensity. In the middle panels (d–f), free‐running FL3‐A rhythms over four subjective days of cells entrained as in (a) and subsequently exposed to LL of 5 μmol photons m^−2^ s^−1^ (d, top), 17 μmol photons m^−2^ s^−1^ (d, middle) and 25 μmol photons m^−2^ (d, bottom); (e) cells grown as in (b) then exposed to LL at 50 (e, top) or to 75 μmol photons m^−2^ s^−1^ (e, bottom). In (f), cells were entrained as in (a) and then exposed to LL of 17 μmol photons m^−2^ s^−1^ at 14°C (top) and 20°C (bottom). (g) Cell division per day calculated in the different experiments (*n* ≥ 3). Statistical analysis of rhythmicity (h), indicating the fraction of the biological replica (*n* ≥ 3) found rhythmic by the Enright Periodogram (EPR) algorithm test and so analysed with fast Fourier transform non‐linear least square algorithm (FFT‐NLLS). (i) Relative amplitude of Error (RAE) of the FFT‐NLLS method fit. In (g, i) solid line indicates the mean and error bars the SD. Fluorescence profiles were baseline detrended and then normalized between 1 and −1 for graphic representation. Coloured envelopes represent SD. White and grey regions represent light and dark periods, grey dashed regions represent subjective nights in LL. ZT indicates the ‘Zeitgeber time’ in LD regime. In LL, the time is counted starting from the end of the last LD day.

Analysis of rhythmicity in *P. tricornutum* was conducted by using as circadian output the red fluorescence per cell (FL3‐A parameter in the flow cytometer) (Annunziata *et al*., 2019). First, we analysed rhythmicity in cells adapted to 16 h : 8 h, LD  under moderate light intensity (25 μmol photons m^−2^ s^−1^) resulting in *c*. 1 cell division per day (Fig. 1a,g; Table S2). FL3 displayed strong diel oscillations with an acrophase of fluorescence *c*. 13 h after light onset (ZT13), hence anticipating by 3 h the transition to darkness (Table S2). During the dark period, fluorescence decreased and reached a minimum at the end of the night, when cells are expected to have completed their cell division. The minimum of fluorescence coincided with the peak of cells in G1 phase (Fig. S2; Annunziata *et al*., 2019), confirming that FL3 serves as a proxy not only for Chl*a* content but also for cell division. When grown under 16 h : 8 h, LD with higher light intensity (75 μmol photons m^−2^ s^−1^), cells showed strong rhythms (Fig. 1b), as for cells experiencing moderate light, with a slightly delayed acrophase of fluorescence (ZT14) and higher growth (Fig. 1g; Table S2).

LD cycles with a photoperiod other than 24 h (also called *T* cycles) are a convenient means for disentangle the effect of external cues and circadian clock on physiological rhythms since the circadian clock is usually not entrained below *T* = 16 h (Roenneberg *et al*., 2005). We thus performed additional experiments by shifting cells adapted to 12 h : 12 h, LD at 25 μmol photons m^−2^ s^−1^ to 6 h : 6 h, LD cycles (period, *T* = 12 h). The analysis in Fig. 1(c) showed the FL3 trend continued to rise during the first 12 h, as for cells adapted to a 24‐h period. However, a decrease in FL3 was already observed after the first 6 h of darkness and, from the second day onwards, FL3 oscillations completely shifted to shorter T12 cycles. Since this coincided with a decrease in growth (0.5 divisions d^−1^) (Fig. 1g; Table S2), it is likely that the observed ultradian fluorescence patterns primarily reflect the inhibitory effect of anticipated dark on the cell cycle. To measure circadian rhythms, we thus focused subsequent analyses in LD entrained cells exposed to free‐running conditions, where the contribution of other processes (e.g. cell cycle and direct responses to environmental changes) should be minimized.

We first analyzed responses of cells adapted to moderate 16 h : 8 h, LD cycles as in Fig. 1(a) and then exposed to either continuous dark (DD) (Table S2) or continuous white light (LL) of various intensities for 4 d (Fig. 1d). The transition to DD resulted in total flattening of fluorescence signal after the first subjective night (Table S2), as expected upon cell division arrest in darkness (Huysman *et al*., 2010). When transferred to LL under low light intensity (5 μmol photons m^−2^ s^−1^; Fig. 1d, top panel), cells continued to divide over the whole 4‐d period, although at a strongly reduced rate when compared to higher light intensities (Fig. 1g) and the FL3 rhythmicity dampened (Table S2). None of the biological replicates appeared as rhythmic based on our circadian periodicity test (Fig. 1h; Table S2). In the case of cultures exposed to 17 μmol photons m^−2^ s^−1^ LL (Fig. 1d, middle), and thus experiencing during the 24 h a total irradiance approximately equivalent to that previously experienced in 16 h : 8 h, LD cycles, the oscillations showed a robust rhythmic pattern. They persisted for at least 4 d in LL, although with a longer period as compared to LD (*c*. 28 h; Table S2). FL3 oscillations also persisted when the light intensity was maintained at 25 μmol photons m^−2^ s^−1^ in LD and LL (Fig. 1d, bottom; Table S2).

When cells were acclimated to 16 h : 8 h, LD cycles under higher light intensity (75 μmol photons m^−2^ s^−1^) as in Fig. 1(b) and then transferred to LL of 50 μmol photons m^−2^ s^−1^ (Fig. 1e, top; Table S2), oscillations were visible though only during the first 2 d of free‐run, with the amplitude decreasing and the noise in the period calculation increasing subsequently (Fig. 1h,i; Table S2). The rhythmic profiles became even more perturbated in cells exposed to a higher light intensity of 75 μmol photons m^−2^ s^−1^ in LL (Fig. 1e,h,i).

A typical circadian clock feature is the ability to maintain rhythms under a wide range of temperatures. Therefore, we also tested FL3 responses in cells adapted to 16 h : 8 h, LD at  25 μmol photons m^−2^ s^−1^ and the standard growth conditions of 18°C, before transferring them to LL with temperature ranging from 14°C to 22°C (Figs 1f, S3). *Phaeodactylum tricornutum* showed robust circadian rhythms at all tested temperatures below 18°C, with slightly shorter periods at 14°C and 16°C. Similar rhythms were detectable at 18°C and 20°C, but were significantly disrupted at higher temperatures (Fig. S3). When detectable, they showed reduced amplitudes.

Since temperature cycles have been reported to entrain the circadian clock (Rensing & Ruoff, 2002), we also investigated the combined role of light and temperature cycles on *P. tricornutum* rhythms. To this end, cells were grown in 16 h : 8 h, LD cycles at 25 μmol photons m^−2^ s^−1^, using 18°C as temperature during the L period and 14°C during the D period for 3 wk. Afterward, cells were transferred to LL at 17 μmol photons m^−2^ s^−1^ and 18°C (Fig. S4). This mild temperature gradient has been chosen based on the experiment in Fig. S3, to avoid perturbation of higher temperatures on FL3 rhythms. The rhythms persisted for a similar number of days in free‐running conditions (Fig. S4) as those observed in cells entrained by LD cycles alone (Fig. 1d, middle), albeit with a slightly shorter period. This suggests that, under the conditions tested, the addition of temperature cycles to the LD cycles has a minimal effect on circadian clock activity.

Taken together, these experiments supported the presence of a circadian oscillator in diatoms and identified the experimental conditions for analysing its activity and consequence of its perturbation in *P. tricornutum RITMO1* mutants.

### *RITMO1* affects fluorescence rhythmicity under free‐running conditions

*RITMO*1 gene editing was achieved by the proteolistic bombardment of the CRISPR‐Cas9 protein (Serif *et al*., 2018). Sequence analyses confirmed the mutagenesis of *RITMO1* by biallelic frameshift mutations in two lines, defined as KO1 and KO2 (Fig. S5). The *P. tricornutum* WT, the two knock‐out (KO) lines, a transgenic line not presenting any mutation for *RITMO1* (CTR) and the previously generated *RITMO1* OE1 (Annunziata *et al*., 2019) were analysed for their fluorescence rhythms.

**Fig. 2:**
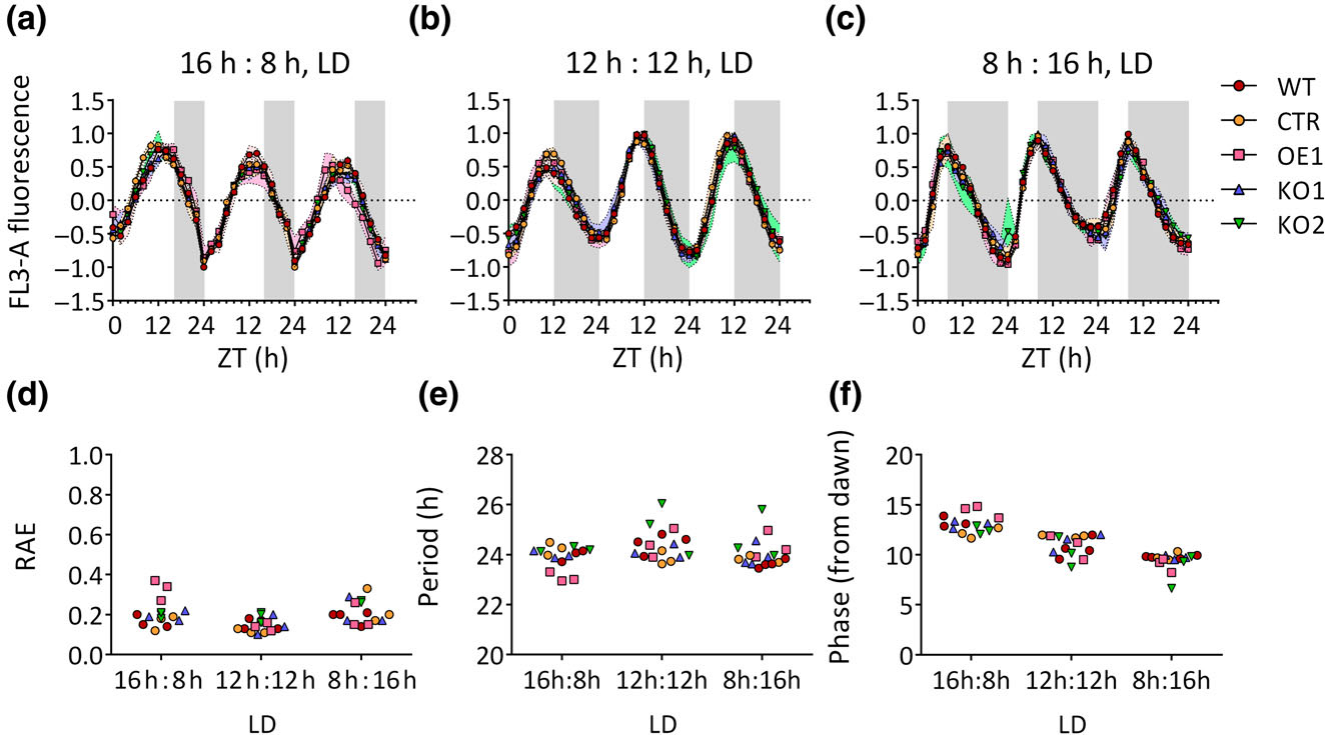
Analysis of the effect of the *RITMO1* expression perturbation on *Phaeodactylum tricornutum* cellular fluorescence rhythmicity under light : dark (LD) cycles. Cellular fluorescence profiles (FL3‐A parameter) of the *P. tricornutum* wild‐type (WT), the transgenic control (CTR), the ectopic *RITMO1* overexpression (OE1) and knock‐out (KO1 and KO2) lines adapted to 25 μmol photons m^−2^ s^−1^ under different photoperiods: 16 h : 8 h (a); 12 h : 12 h (b); 8 h : 16 h (c) LD cycles, (*n* ≥ 3). Relative amplitude of error (RAE) of the FFT‐NLLS (fast Fourier transform non‐linear least square algorithm) method fit (d); Period (e) and Phase estimation (relative to dawn) (f) calculated with FFT‐NLLS algorithm. Fluorescence profiles were baseline detrended and then normalized between 1 and −1 for graphic representation. Coloured envelopes represent SD. White and grey regions represent light and dark periods.

We first tested the effect of *RITMO1* KOs and overexpression in cells exposed to LD cycles of moderate intensity (25 μmol photons m^−2^ s^−1^) under various photoperiods (Fig. 2). Strong fluorescence oscillations were observed in all the lines and conditions tested (Figs 2, S6; Table S3), for cultures exhibiting between 0.8 division d^−1^ (under 8 h : 16 h, LD, and 12 h : 12 h, LD; Table S3) and 1 division d^−1^ (under 16 h : 8 h, LD). *RITMO1* KO and OE lines showed rhythmic fluorescence trends comparable to those of the WT and CTR lines (Figs 2, S6; Table S3).

**Fig. 3:**
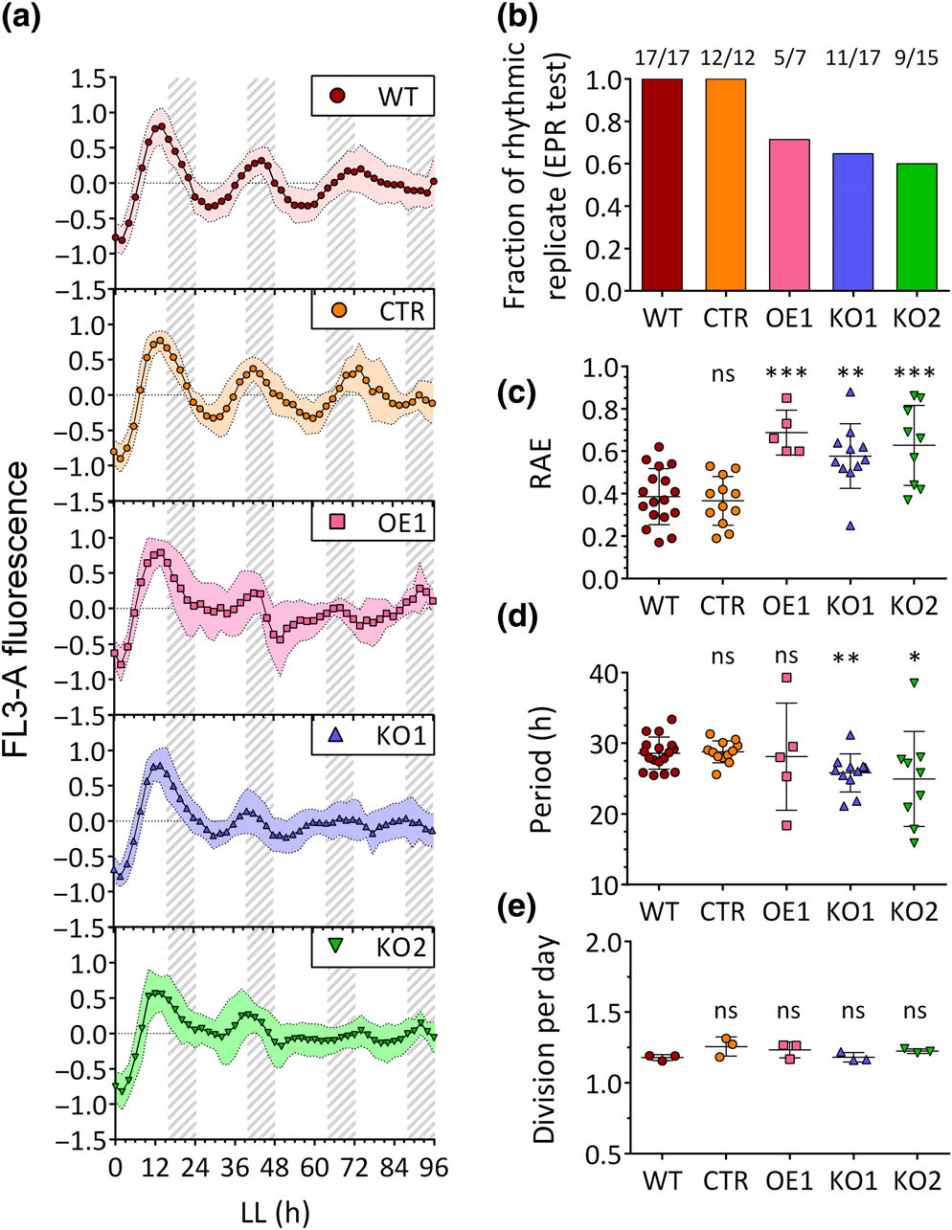
Analysis of the effect of the *RITMO1* expression perturbation on *Phaeodactylum tricornutum* circadian cellular fluorescence rhythmicity. (a) Fluorescence profile (FL3‐A parameter) of the *P. tricornutum* wild‐type (WT), the transgenic control (CTR), the ectopic *RITMO1* overexpression (OE1) and knock‐out (KO1 and KO2) lines adapted to 16 h : 8 h, light : dark (LD) cycles, 25 μmol photons m^−2^ s^−1^ and subsequently exposed to LL 17 μmol photons m^−2^ s^−1^ (*n* ≥ 7). (b) Fraction of replicates that passes the Enright Periodogram (EPR) algorithm test for all strains. Further analyses are only performed for the lines found rhythmic with EPR test: (c) Relative amplitude of error (RAE) of the FFT‐NLLS (fast Fourier transform non‐linear least square algorithm) method fit; (d) Period estimation obtained with FFT‐NLLS method; (e) Division per day along the experiment (*n* = 3). Solid lines indicate the mean and error bars the SD. Fluorescence profiles were baseline detrended and then normalized between 1 and −1 for graphic representation. Coloured envelopes represent SD. Statistical differences were examined using unpaired Student's *t*‐test with the WT as reference sample (*, *P* < 0.05; **, *P* < 0.01;***, *P* < 0.001; ns, non significant). Grey dashed regions represent subjective nights in free running LL conditions. In LL, the time is counted starting from the end of the last LD day.

We then examined circadian rhythms in WT, CTR, *RITMO1* KO and OE lines following a shift from LD to LL (Fig. 3) applying the previously established conditions for detection of the circadian clock (25 LD to 17 LL μmol photons m^−2^ s^−1^). Strongly altered FL3 rhythmicity was observed in OE1 (Fig. 3; Table S4) in LL, confirming results obtained previously under constant blue light (Annunziata *et al*., 2019). Here, we showed that rhythmicity was also significantly affected in the KO lines (Fig. 3; Table S4). In cases where we were able to detect rhythms in KO1 and KO2 in LL; however, they showed a reduced amplitude, when compared to WT and CTR, which is paralleled by an RAE increase (Fig. 3b–e; Table S4). Growth analysis of the different cell lines (Figs 3e, S7; Table S4) showed a comparable growth, excluding that the alterations of circadian FL3 rhythmicity observed in RITMO1 KO and OE1 are attributable to growth defects. In addition, both KO lines and the OE line showed a similar alteration of rhythmicity under higher LL light intensity (25 LD to 25 LL μmol photons m^−2^ s^−1^), while clock activity was still detectable in the WT and CTR strains (Fig. S8).

**Fig. 4:**
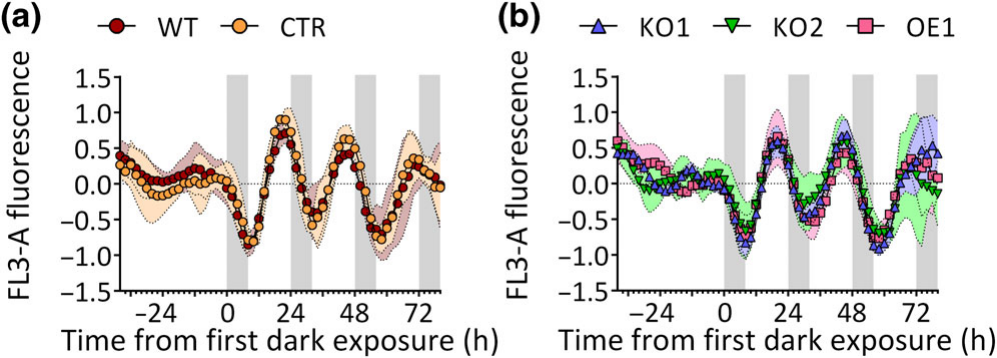
Re‐entrainment of *Phaeodactylum tricornutum* WT and transgenic lines with light : dark (LD) cycles. Cellular fluorescence (FL3‐A parameter) rhythmicity of the *P. tricornutum* wild‐type (WT) and the transgenic control (CTR) line (a), the *RITMO1* ectopic overexpression (OE1) and knock‐out (KO1 and KO2) lines (b) re‐exposed to 16 h : 8 h, LD following constant light desynchronisation (*n* ≥ 3). Fluorescence profiles were baseline detrended and then normalized between 1 and −1 for graphic representation. Coloured envelopes represent SD. White and grey regions represent light and dark periods.

We further inquired whether alteration of *RITMO1* expression influences the capacity of the cells to dynamically respond to environmental inputs. To do so, non‐synchronized cells grown in continuous light for 2 wk were exposed to 16 h : 8 h, LD photoperiod (Fig. 4), in a mirror treatment to that reported in Fig. 3. All the lines recovered 24 h rhythmicity already after the first night and no phase shift was observed, indicating that a treatment of 8 h of darkness is sufficient to re‐synchronize the population and that RITMO1 is not essential for photoperiodic entrainment of the FL3 rhythms.

### RITMO1 is implicated in the circadian regulation of photosynthesis

**Fig. 5:**
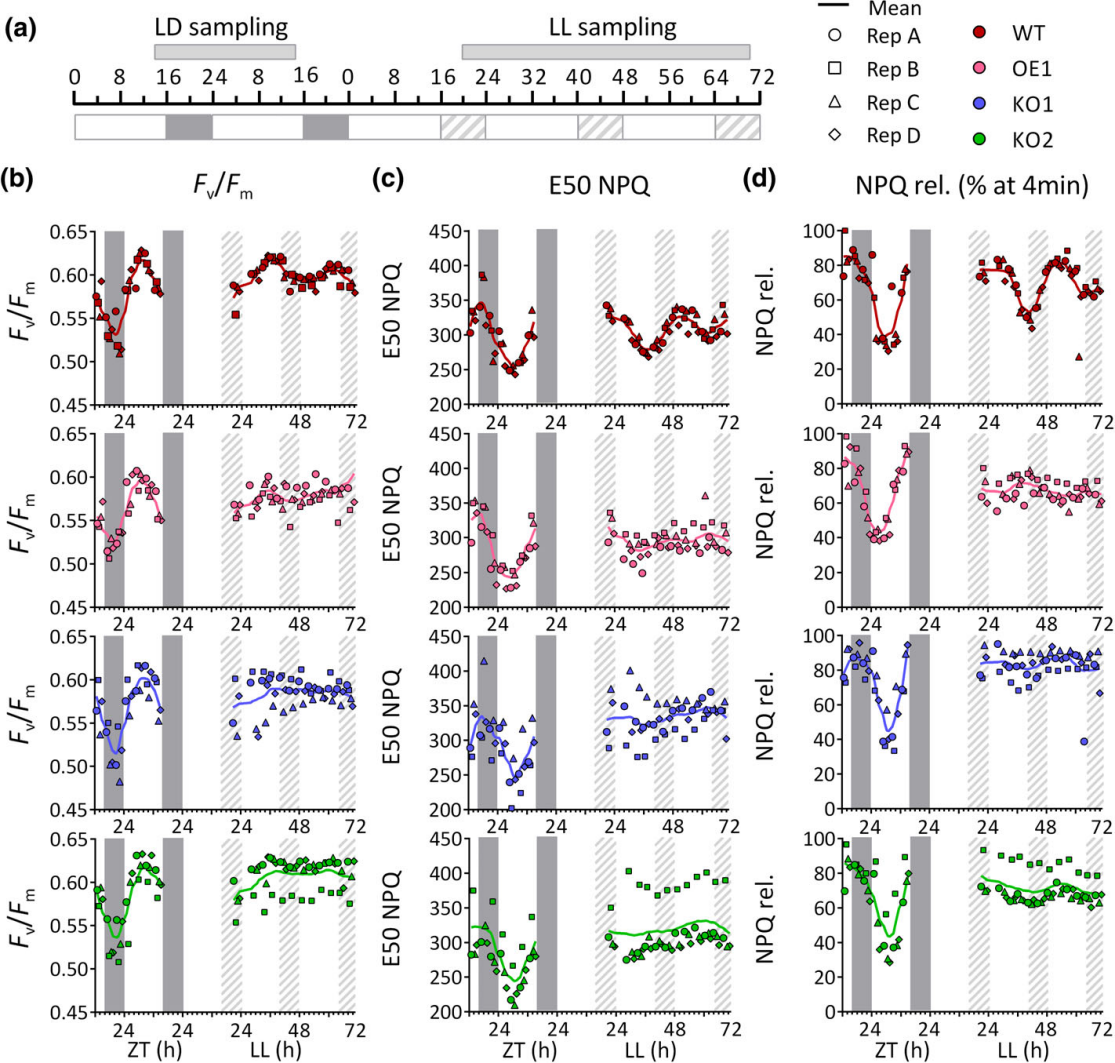
Analysis of the effect of *RITMO1* ectopic overexpression and knock‐out on different *Phaeodactylum tricornutum* photosynthetic parameters. (a) Schematic representation of sampling times for the wild‐type (WT), *RITMO1* knock‐out (KO1 and KO2) and ectopic overexpression (OE1) lines grown under 16 h : 8 h, light : dark (LD) cycles, 50 μmol photons m^−2^ s^−1^ and subsequently exposed to LL 30 μmol photons m^−2^ s^−1^. Samples were collected continuously and measured one‐by‐one in the following order WT, KO1, KO2, OE1 from replicate A to replicate D, with 15 min interval due to the measurement. Measurements were performed over 24 h in LD and 48 h in LL. In free‐running, samples were collected starting at LL 16. (b) Maximum dark‐acclimated quantum yield of PSII (*F*_v_/*F*_m_), (c) E50NPQ, light intensity where 50% of the maximum NPQ is reached and (d) NPQ relaxation (NPQ rel.), percentage of NPQ at maximum light intensity relaxed after 4 min in the dark. Dots, squares, triangles and diamonds represents the individual measures for biological replicates A–D, lines represent the moving average of all replicates (window of 4 h). White and grey regions represent light and dark periods, grey dashed regions represent subjective nights in free‐running conditions. ZT indicates the ‘Zeitgeber time’ in LD regime.

The observed rhythmic patterns of FL3 in free‐running conditions could reflect rhythmic patterns in plastid content or/and activity, for example, the number of PSIIs per cell or in the number of Chl molecules per PSII (i.e. related to the functional absorption cross‐section of PSII, σPSII). This prompted us to monitor several photosynthetic parameters with PSII fluorescence (the Materials and Methods section; Methods S2), comprising four groups of processes: (i) PSII integrity and light absorption capacity (*F*_v_/*F*_m_ and σPSII), (ii) photosynthetic electron transport, (iii) maximal photoprotection capacity (NPQ_m_), and (iv) kinetics and regulation of NPQ (E50NPQ and NPQrel), the latter in *P. tricornutum* being strictly mediated by the xanthophyll cycle (XC) (Blommaert *et al*., 2021). This analysis was first carried out on 16 h : 8 h, LD adapted cells, where all measured parameters showed clear diel oscillations (Figs 5, S9; Table S5). Cells were then transferred to LL, and the photophysiology analysis was resumed on the second and the third days (avoiding transfer‐related transient responses in the first 24 h) (Fig. 5a). The most pronounced rhythms in the WT (*P* < 0.01; Table S5) were found for the two observables informing on the dynamic regulation of the XC, through the light‐dependence (E50NPQ) and kinetics in darkness (NPQrel.) of NPQ, whereas a significant rhythmic behavior for *F*_v_/*F*_m_ was also observed (Fig. 5; Table S5). In the case of RITMO1 OE and KOs, values of these three parameters showed high dispersion between replicates and no rhythms were detected by statistical tests in LL. In the case of OE lines, a slight phase anticipation effect was observed for NPQ dynamics parameters in LD.

### RITMO1 plays a central role in the circadian control of gene expression in diatoms

**Fig. 6:**
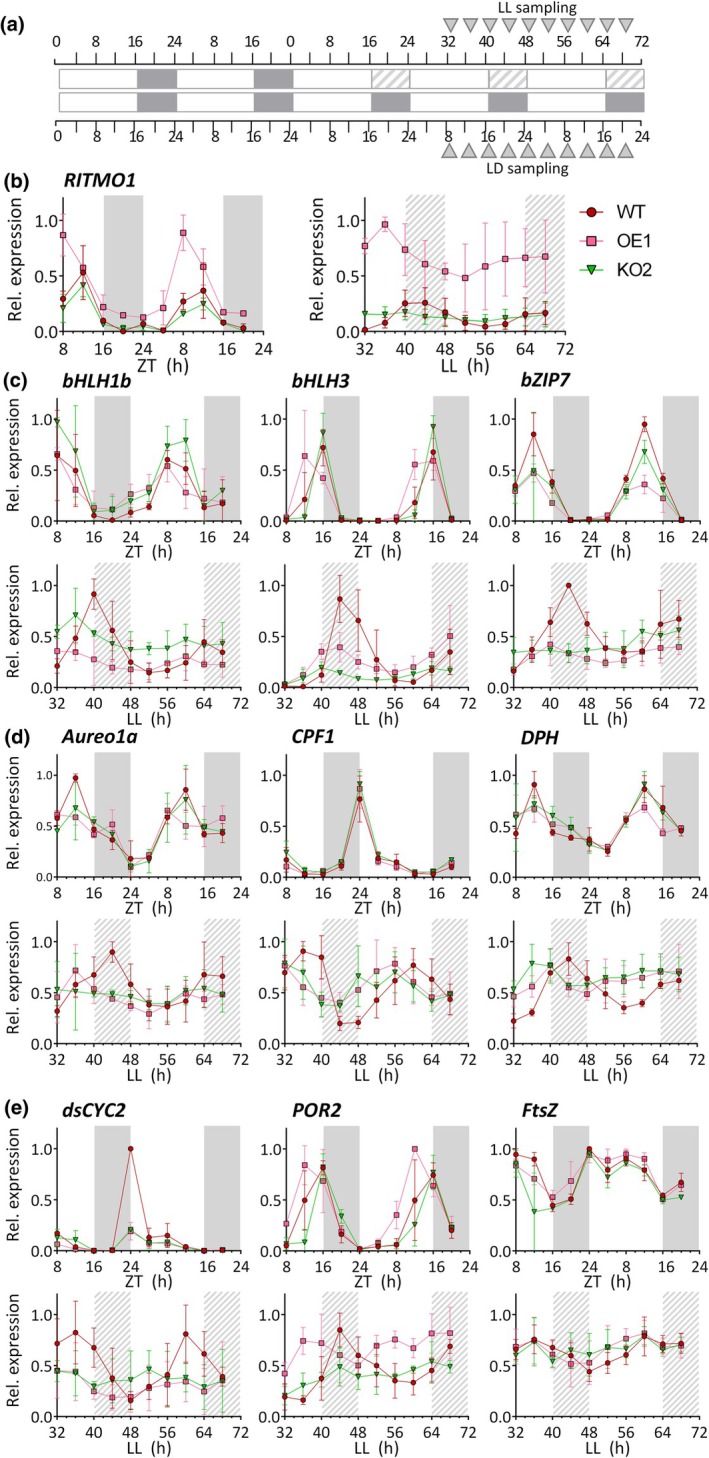
Analysis of the effect of *RITMO1* knock‐out and ectopic overexpression on *Phaeodactylum tricornutum* gene expression rhythmicity. (a) Schematic representation of the sampling points for the wild‐type (WT), the *RITMO1* ectopic overexpression (OE1) and knock‐out (KO2) lines adapted to 16 h : 8 h, light : dark (LD) cycles at 25 μmol photons m^−2^ s^−1^ and then exposed to LL 17 μmol photons m^−2^ s^−1^. Samples were collected every 4 h over 36 h in parallel from cells adapted to LD and from LL. In free running, samples were collected starting at LL 32. Analyses by qRT‐PCR of selected genes in WT, KO2 and OE1 lines under LD and LL cycles. (b) Expression profiles of endogenous and ectopic *RITMO1* transcripts; (c) Transcription factors *bHLH1b*, *bHLH3* and *bZIP7*; (d) Diatom phytochrome (*DPH*), Cryptochrome Photolyase Family 1 (*CPF1*) and Aureochrome 1a (*AUREO1a*) photoreceptors and (e) cyclin *dsCYC2*, protochlorophyllide oxidoreductases 2 (*POR2*), and the filamenting temperature‐sensitive (*FtsZ*) genes. Expression values represent the average of three technical replicates for biological replicates (*n* = 2 in LD and *n* = 3 in LL) ± SD, normalized using the *RPS* and *TBP* reference genes. Expression values are given relative to the maximum expression for each gene, where ‘1’ represents the highest expression value of the time series. White and grey regions represent light and dark periods, grey dashed regions represent subjective nights in free‐run conditions. ZT indicates the ‘Zeitgeber time’ in  LD regime.

Gene expression of a set of genes by RT‐qPCR was measured in WT, OE1 and KO2 lines every 4 h for 2 d in LD and in LL. In free‐running, sampling was performed between LL32 and LL68, corresponding to the second and third subjective night and day, as in the photosynthesis experiment (Fig. 6a). *RITMO1* showed rhythmic expression patterns in LD, which are maintained in LL in the WT, although with a delayed peak of expression (Fig. 6b). As previously reported (Annunziata *et al*., 2019), the OE1 line showed higher *RITMO1* expression levels and anticipation of the maximum of expression compared to the WT in LD (Fig. 6b), due to the overexpression of *RITMO1* driven by the *FcpB* promoter. In LL, OE1 displayed higher expression level than WT and a distinct rhythmic pattern, with an acrophase during the subjective light period. For KO2, an expression pattern similar to the WT was observed for *RITMO1* in LD, while loss of circadian expression was observed in LL. This indicated that the CRISPR‐Cas mutagenesis did not significantly alter *RITMO1* mRNA expression in LD, but generated a non‐functional protein (Fig. S5), likely perturbating a circadian autoregulatory feedback loop. We also analysed other TFs transcripts (*bHLH1b*, *bHLH3* and *bZIP7*) exhibiting strong abundance rhythmicity in LD cycles (Annunziata *et al*., 2019). Expression peak anticipation was observed for *bHLH3* in LD in the OE1 only (Fig. 6c). Rhythms were maintained in LL in WT cells (Fig. 6c), but they were strongly altered in both OE1 and KO2 for the *bHLH1b* and *bZIP7* transcripts. For *bHLH3*, a damped rhythmicity was still detected in KO2, although with an altered phase.

We next analysed the expression of diatom photoreceptors, potential actors in the light input pathway to the clock (Fig. 6d). Rhythmic expression was observed for the phytochrome photoreceptor (*DPH*) (Fortunato *et al*., 2016; Duchêne *et al*., 2025) in LD and at the second and third day in LL in WT. Only minor residual oscillations were observed in OE1 and KO2 at the second subjective day, compared to the WT. Rhythmic patterns in LD and at the second and third LL period were also detected for the blue‐light receptor *CPF1*. KO2 and OE1 showed no alteration in LD compared to WT, but a temporal shift when compared with WT in LL. The pattern of the *AUREO1a* transcript was particularly interesting since this blue light receptor also acts as a TF controlling the expression of an important number of genes in *P. tricornutum*, including *RITMO1* under LD and DD conditions (Madhuri *et al*., 2024). The *AUREO1a* rhythmic expression pattern observed under LD and LL conditions in the WT was altered in *RITMO1* KO2 and OE1 in LL, suggesting that RITMO1 and AUREO1a might be part of a same regulatory system. This hypothesis is further supported by the altered circadian FL3 fluorescence rhythmicity observed in *AUREO1a* KO mutants, compared to WT cells in LL (Fig. S10).

We then investigated the expression of putative clock regulated genes (Fig. 6e). The cell cycle regulator *dsCYC2*, whose expression is strongly and transiently induced after switching from darkness to light (Huysman *et al*., 2010, 2013) showed a rhythmic pattern in the WT in LD, with an increase in the expression before the light onset (Fig. 6e), which is lost in OE1 and KO2. In LL, *dsCYC2* rhythmic expression was observed for WT, but not for the OE1 and KO2 (Fig. 6e). The *CYCP6* gene, whose expression has been shown to be associated with the G1 phase, had a rhythmic expression in LD, with a peak at the end of the night, in all the analysed strains. Minor rhythmicity was observed in WT in the second free‐running day, further reduced at the third day. Rhythmic patterns were not observed in the transgenic KO and OE lines for *CYCP6* (Fig. S11). The *CYCB1* gene, a marker of the G2/M phase, showed a clear rhythmic pattern in LD, peaking at the end of the light period, which was overall unaltered in KO2 and OE1. However, the expression of *CYCB1* appeared arrhythmic in LL in all the lines (Fig. S11).

We also analysed the expression patterns of two genes linked to chloroplast activity: *FtsZ*, encoding a putative component of the diatom plastid division machinery (Gillard *et al*., 2008; TerBush *et al*., 2013) and *POR2* (protochlorophyllide oxidoreductase 2) involved in Chl biosynthesis (Hunsperger *et al*., 2016; Fig. 6e). For *POR2*, we observed rhythms in both LD and LL. Gene expression anticipation was observed for the OE in LD, while deregulation of rhythmic pattern was observed for both OE and KO in LL. On the contrary, for *FtsZ* we observed rhythmic oscillation in LD, but not in LL, even for the WT. Interestingly the expression pattern is not altered in *RITMO1* OE or KO lines, ruling out the possible regulation of *FtsZ* by an endogenous oscillator.

### RITMO1 mutation complementation rescues circadian rhythms in *P. tricornutum*

**Fig. 7:**
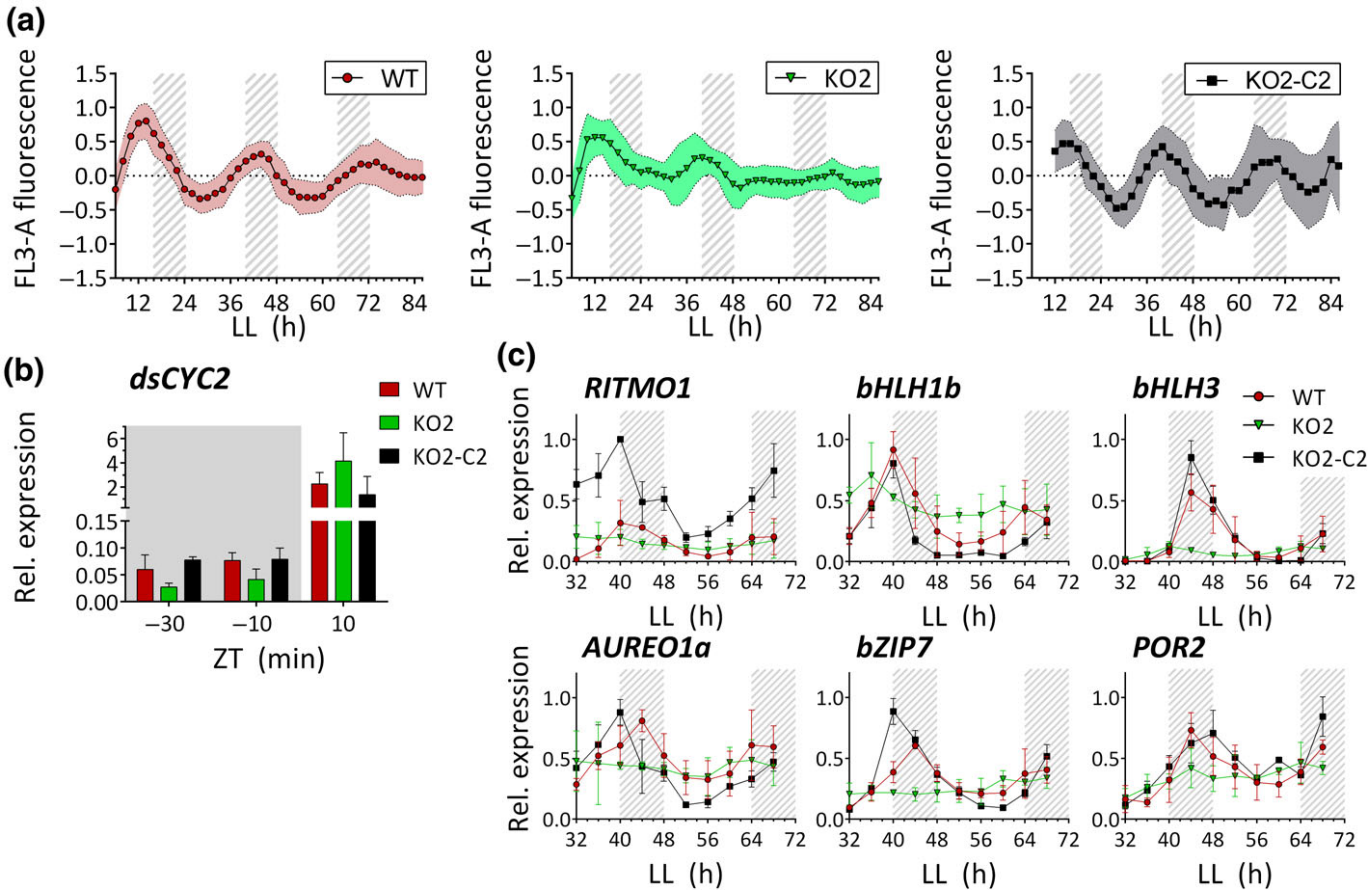
Analysis of rhythmicity in the *Phaeodactylum tricornutum RITMO1* knock‐out strain (KO2) transformed with the RITMO1p::RITMO1g:Venus::RITMO1t construct to obtain the KO2‐C2 complemented line. (a) Normalised and baseline detrended cellular FL3‐A fluorescence profiles in free run from *Phaeodactylum tricornutum* wild‐type (WT), *RITMO1* KO2 and complemented KO2‐C2 strains (*n* ≥ 6). Cells were entrained under 16 h : 8 h, light : dark (LD) cycles, 25 μmol photons m^−2^ s^−1^ before the shift to LL 17 μmol photons m^−2^ s^−1^. (b) Analysis by qRT‐PCR of *dsCYC2* relative expression in the same strains grown under 16 h : 8 h, LD at 25 μmol photons m^−2^ s^−1^ and collected for the analysis before and after the light onset at the time indicated. Bars represent mean ± SD expression values normalised using *RPS* and *TBP* reference genes. (c) Analyses by qRT‐PCR in LL of the expression profiles of the *RITMO1*, *bHLH1b*, *bHLH3*, *AUREO1a*, *bZIP7* and *POR2* genes in WT, KO2 and KO2‐C2 lines. Mean ± SD gene expression values normalised using *RPS* and *TBP* reference genes and relative to maximum expression, ‘1’ representing the maximum of expression across the time series. Expression values represents the average of three technical replica for each biological replica (*n* = 3). White and grey regions represent light and dark periods, grey dashed regions represent subjective nights in LL. ZT indicates the “Zeitgeber time” in LD regime. In LL , the time is counted starting from the end of the last LD day.

To further prove the involvement of RITMO1 in circadian rhythms, we complemented the KO2 line with a construct containing the genomic *RITMO1* sequence fused to the *Venus* reporter under the control of *RITMO1* promoter and terminator sequences (Methods S1; Fig. S12). Different transgenic lines were obtained and screened by Western blot by using anti‐GFP antibody to check transgene expression time and levels at the beginning and end of the light period (Fig. S13a). Nuclear localisation was confirmed for these lines by fluorescence microscopy (Fig. S13b). However, when we analysed FL3 rhythms, only the KO2‐C2 line, which displays moderate and rhythmic expression of RITMO1 (Fig. S13a), rescued the WT circadian patterns (Fig. 7a). We further use this line to analyse gene expression rhythms. A specific feature of the circadian clock is the capacity to anticipate periodic LD changes. In Fig. 6 we observed lower expression of the cell cycle regulator *dsCYC2* in KO and OE, compared to WT, at the D to L transition. Therefore, we analysed *dsCYC2* expression with a higher temporal resolution before and after the light onset (Fig. 7b). The analysis indicated a delay in the light anticipation response and in the rapid‐light induced expression in KO2 compared with WT (Fig. 7b) that was rescued in the KO2‐C2 line. Analyses of selected genes in LL also confirmed a recovery of rhythmic expression pattern in free run for all the analysed genes. For some genes, such as *AUREO1a* and *bZIP7* we observed an anticipation of the expression time in KO2‐C2, and for *dsCYC2* (Fig. S14), a reduced amplitude. These phenotypes could be linked to a partial deregulation of the regulatory loop involving RITMO1 due to the gene overexpression in the KO2‐C2 line compared to the WT (Fig. 7c) or deregulation of still uncharacterized RITMO1 interactors.

## Discussion

The characterisation of various biological rhythms in this study reveals a complex regulation of diatom responses to light and dark cycles, involving both environmental response pathways and internal biological programs. This was established by defining the appropriate experimental conditions to monitor the circadian clock activity (Figs 1, 2, S2–S4) and by analysing how the perturbation of RITMO1 expression affects different rhythmic processes. Even if it is not yet possible to assess whether RITMO1 is a component of the central oscillator or a slave oscillator transducing the master clock's impulses to a subset of circadian‐regulated processes (e.g. Heintzen *et al*., 1997), our data unambiguously supports a role of this protein in a circadian regulatory loop generating rhythms. A strong perturbation of various circadian rhythms is observed upon knock‐out of the single *RITMO1* gene (Figs 3, 5, 6), and these phenotypes are rescued through genetic complementation (Fig. 7). RITMO1 not only regulates its own expression, but also influences that of other clock regulated genes, and it also plays a role in light anticipation responses (Fig. 7). Additionally, altered rhythmicity is observed for several genes under LD cycles in the OE lines. Notably, circadian rhythms in KO lines are rescued only in the line showing the correct expression time and moderate gene overexpression compared to the WT. This suggests that the misregulation of RITMO1 may have a stronger effect than gene obliteration on the transcriptional feedback loops generating rhythms, as reported for OtCCA1 (Corellou *et al*., 2009).

Among the RITMO1 regulated genes, several potential clock components have been identified (Fig. S15). Other TF members of the bHLH family (*bHLH1b* and *bHLH3*), as well as *bZIP7*, represent direct or indirect RITMO1‐target genes. A molecular interplay in circadian regulation can now be proposed for AUREO1a and RITMO1, two TFs that show similar expression timing, peaking at the end of the light period. *RITMO1* expression is regulated by *AUREO1a* (Mann *et al*., 2020; Madhuri *et al*., 2024) and we have shown here that the circadian expression of *AUREO1a* is altered in *RITMO1* mutants, both factors being essential for maintaining circadian fluorescence rhythmicity (Figs 3, S10). The rapid re‐entrainment of biological rhythms in both desynchronized WT and *RITMO1* mutants upon re‐exposure to light dark cycles suggests that RITMO1 is not essential for clock entrainment (Fig. 4). However, this function is expected for AUREO1a due to its additional function as a blue light photoreceptor. The circadian regulated photoreceptors Phytochrome DPH and the blue light Cryptochrome Photolyase 1 CPF1 are also potential candidates of the diatom input pathways (Fig. S15). Further investigations under different fluence rate/quality of light and phase response curves will be needed to assess their role in the photic control of circadian rhythms.

Here, we demonstrate for the first time in diatoms, the involvement of a circadian clock component in the regulation of photosynthesis, as previously observed in plants (Dodd *et al*., 2005). This adds molecular and physiological insights to previous observations based on analyses of circadian carbon uptake, pigment content or gene expression in diatoms (Palmer *et al*., 1964; Ragni & d'Alcalà, 2007; Su *et al*., 2024). Parameters informing on photosynthetic electron transport (maximal capacity under saturating light or performance under light‐limiting regime) or photoprotection capacity (NPQ_m_) show diel cycles but do not oscillate significantly under free‐running conditions (Fig. S9; Table S5). Although we cannot rule out that these parameters display oscillatory patterns below the sensitivity of our methodological approach, these rhythms are clearly smaller than those linked to PSII light absorption and conversion (*F*_v_/*F*_m_ and σPSII) and especially to the regulation of NPQ (E50NPQ and NPQrel.), which are under RITMO1‐dependent circadian control. Many known rhythmic processes can influence *F*_v_/*F*_m_, such as PSII recycling (Li *et al*., 2016) or NPQ relaxation (this study). The circadian regulation of Chl biosynthesis (Ragni & d'Alcalà, 2007; Hunsperger *et al*., 2016), here supported by RITMO1 regulation of the *POR2* gene (Figs 6, 7), could influence circadian rhythms of σPSII.

Interestingly, the photosynthetic parameters most strongly regulated by the circadian clock are those informing on the dynamics of fast NPQ regulation. The ecological success of diatoms in turbulent waters is often attributed to their efficient photoprotective capacity via NPQ (Lavaud *et al*., 2007), which allows the avoidance of photodamages under high light, while being rapidly tuned down to avoid ‘energy wasting’ when irradiance decreases. Therefore, the clock regulation of NPQ dynamics may contribute in the real‐time adjustment/anticipation of photoprotective mechanisms to the light environment and time of the day. The lack of persistence of oscillations in the maximal NPQ capacity (NPQ_m_) in free‐running makes it unlikely that the concentration of the main NPQ molecular actors, the Lhcx proteins and the XC pigments (Buck *et al*., 2021; Croteau *et al*., 2025), is a primary target of the circadian clock. At the contrary, the circadian regulated E50NPQ and NPQrel parameters only depend on the relative rates of the diadinoxanthin de‐epoxidase (increasing NPQ) and diatoxanthin epoxidase (decreasing NPQ) enzymes of the XC (Blommaert *et al*., 2021). As some XC genes maintain rhythmic expression in the dark (Annunziata *et al*., 2019), RITMO1 could contribute to circadian regulation or to post‐translational modification of these enzymes. Alternatively, RITMO1 could regulate the availability of co‐substrates of the XC enzymes, luminal ascorbate for diadinoxanthin de‐epoxidase or stromal NADPH for diatoxanthin epoxidase, which in turn, could impose circadian rhythms in the kinetic rates of these enzymes, regardless of their quantity. Circadian rhythms of NADPH levels are known in *A. thaliana*, as well as in ascorbate pathway genes in plants (Dowdle *et al*., 2007) and *Euglena gracilis* (Kiyota *et al*., 2006). The anticipation of NPQrel. observed in OE1 line in LD compared to WT and KO lines (Fig. 5d) suggest that even under enforced photoperiodic rhythms, RITMO1 accumulation may modulate the activities of the XC enzymes and NPQ dynamics.

The *P. tricornutum RITMO1* mutants show no growth limitation in either LD or in LL. At this stage, we do not know if this is due to the potentially redundant function of other clock proteins (e.g. other bHLH‐PAS), the peripheral role of RITMO1 in the circadian system, or the weak activity of the diatom clock (Bordyugov *et al*., 2015; Poliner *et al*., 2019). In cyanobacteria, the effect of clock mutation on fitness is only clearly seen in competition experiments between circadian period mutants and WT cells and when their internal rhythms match those of the environmental cycle (Ouyang *et al*., 1998; Woelfle *et al*., 2004). Similar observations have been made also with a variety of clock mutants in plants (Dodd *et al*., 2005; Yerushalmi *et al*., 2011). Therefore, further research with multiple clock mutants exposed to different environmental LD cycles will be needed to clearly assess the role of the diatom clock on fitness.

Our analyses also reveal that circadian clock activity in *P. tricornutum* is masked under certain laboratory conditions. Limited persistence of fluorescence rhythmicity in free‐running cells was observed under very low light or at light intensities high enough to promote a more sustained growth (Fig. 1). Similar to what is reported in the green alga *Nannochloropsis oceanica* (Poliner *et al*., 2019), disruption of rhythmicity was also observed when cells were exposed to temperatures > 2°C above their growth temperature (Fig. S3), which probably has an impact on metabolism (Fawley, 1984; Rehder *et al*., 2023). Poor free‐running rhythmicity could result from a loss of rhythmicity or from asynchrony between individual cells within the bulk culture. *De facto*, FL3 is a good proxy of the synchronicity in diatom population as it captures different observables, including cell growth, cell cycle progression (Huysman *et al*., 2010) and plastid activity (Falciatore *et al*., 2022). However, all these processes depend on direct environmental inputs that could override clock activity. Darkness provokes cell cycle arrest in diatoms, which explains the loss of FL3 rhythmicity in DD. Enforced desynchronisation by 6 h : 6 h, LD cycles, resulting in short oscillations occurring twice per circadian cycle, also affects *P. tricornutum* growth (Fig. 1). The gating of the cell cycle by an endogenous oscillator has not been demonstrated in our study, but it is plausible to hypothesize that the circadian clock and the cell cycle co‐act to regulate light‐dependent diatom cell division, as proposed in the green algae (Goto & Johnson, 1995; Moulager *et al*., 2007, 2010) and Nannochloropsis (Braun *et al*., 2014; Poliner *et al*., 2019). Thus, the observed perturbation in the circadian rhythmicity of *P. tricornutum* fluorescence may result from an uncoupling between the circadian regulation of cell division and the direct response to changing environmental cues, similar to what has been observed in *O. tauri* under high light (Moulager *et al*., 2007). Acclimation of photosynthesis may also play a role. In many phototrophs, photosynthesis is a major circadian output, and our data also support its regulation by the clock in diatoms. Evidences indicate that photosynthesis can, in turn, regulate clock activity by acting on input pathways (Farré & Weise, 2012; Haydon *et al*., 2017; de Barros Dantas *et al*., 2023). Therefore, in addition to the cell cycle, a strong interplay may exist between the circadian clock and photophysiology in diatoms to coordinate critical cellular and metabolic processes under specific light conditions. These aspects will deserve further characterisation via the generation of *ad hoc* mutants in distinct light‐regulated processes and extended physiological characterisation, as has been elegantly shown in plants (Haydon *et al*., 2013).

Finally, this study positions marine diatoms as novel experimental model organisms for studying the chronobiology of algae derived from secondary endosymbiosis, which constitute a significant proportion of phytoplankton. The strong perturbation of circadian rhythms observed in *P. tricornutum* at just 4°C above its optimal growth condition, coupled with the absence of clear evidence for temperature clock entrainment under our experimental conditions, raises important questions about the role of this cue in regulating the circadian clock in these algae. In marine environments, many phytoplanktonic species typically experience far less drastic temperature changes during LD cycles compared to terrestrial organisms or marine organisms leaving in intertidal zones. Additionally, diatoms dominate the polar regions (Pierella Karlusich *et al*., 2020), which are characterized by cold temperatures, but also dramatic seasonal changes in photoperiod. The recent discovery of photoperiodism in cyanobacteria (Jabbur *et al*., 2024), along with emerging evidence of photoperiodic regulation of photosynthesis in some diatom polar species (Guérin *et al*., 2024), raises also questions about the potential role of circadian clock in latitudinal and seasonal adaptation, similarly to what has been observed in plants and animals (Nishiwaki‐Ohkawa & Yoshimura, 2016). Therefore, more detailed studies on the effects of varying temperature and light cycles on diatom biological rhythms will provide valuable insights into the functional diversification of circadian systems (Laosuntisuk *et al*., 2023) and, more broadly, into the significance of so far generalized clock properties for life in the oceans (Häfker *et al*., 2023).

## Competing interests

None declared.

## Author contributions

AF coordinated the project and designed research with AM and RM, and with the contribution of J‐PB, BB, MJ, F‐YB and PGK; AM and RM performed most of the analyses, with the support of LC for the temperature entrainment experiments. SCN, LC, AH and MJ contributed to the characterisation of gene expression and complementation of *RITMO1* knock‐out lines. RM, DC and BB characterized photosynthesis features; DJ and FD generated the *RITMO1* knock‐out; NFS characterized the circadian rhythms in *Aureo1a* knock‐out. AF, AM, RM, BB, FD, F‐YB, MJ and DC analysed and interpreted the data; AF, AM and RM wrote the manuscript. All the authors discussed the results and commented on the manuscript. AM and RM contributed equally to this work.

## Disclaimer

The New Phytologist Foundation remains neutral with regard to jurisdictional claims in maps and in any institutional affiliations.

## Supporting information


**Fig. S1** Evolution of non‐photochemical quenching (NPQ) vs light intensity and kinetics of NPQ relaxation in darkness.
**Fig. S2** Characterisation of growth, cellular fluorescence and cell cycle synchronisation in *Phaeodactylum tricornutum* cells (*n* ≥ 3) entrained under 16 h : 8 h, light : dark cycles,  25 μmol photons m^−2^ s^−1^.
**Fig. S3** Characterisation of cellular fluorescence rhythmicity in *Phaeodactylum tricornutum* WT strain entrained under 16 h : 8 h, light : dark cycles, 25 μmol photons m^−2^ s^−1^ at 18°C and after the switch to LL 17 μmol photons m^−2^ s^−1^ at various temperatures.
**Fig. S4** Characterisation of cellular fluorescence rhythmicity of *Phaeodactylum tricornutum* grown under 16 h : 8 h, ligh : dark cycles, and 14°C : 18°C temperature cycles and following a shift to LL free‐running condition.
**Fig. S5** Analysis of *RITMO1* gene modification in knock‐out (KO) lines compared to the wild‐type (WT) sequence.
**Fig. S6** Characterisation of cellular fluorescence rhythmicity of *Phaeodactylum tricornutum* wild‐type (WT), the transgenic control (CTR), the *RITMO1* knock‐out (KO1 and KO2) and ectopic overexpression (OE) lines (*n* ≥ 3) under light : dark (LD) cycles.
**Fig. S7** Analysis of growth capacity in *Phaeodactylum tricornutum* wild‐type (WT), the transgenic control (CTR), the *RITMO1* ectopic overexpression (OE1) and knock‐out (KO1 and KO2) lines.
**Fig. S8** Characterisation of cellular fluorescence rhythmicity in *Phaeodactylum tricornutum* wild‐type (WT), the transgenic control (CTR), the *RITMO1* ectopic overexpression (OE1) and knock‐out (KO1 and KO2) lines entrained at 16 h : 8 h, light : dark (LD) cycles, 25 μmol photons m^−2^ s^−1^ after the switch to LL 25 μmol photons m^−2^ s^−1^.
**Fig. S9** Analysis of photosynthetic parameters in the *Phaeodactylum tricornutum* wild‐type (WT), *RITMO1* ectopic overexpression (OE1) and knock‐out (KO1 and KO2) lines.
**Fig. S10** Characterisation of cellular fluorescence rhythmicity in *Phaeodactylum tricornutum* WT (Pt4) and the *Aureochrome1a* knock‐out (KO8 and KO9) lines under 16 h : 8 h, light : dark (LD), 25 μmol photons m^−2^ s^−1^ entrainment and following a switch to LL of 17 μmol photons m^−2^ s^−1^ for 3 d.
**Fig. S11** Analysis of the rhythmic expression of selected genes by qRT‐PCR in *Phaeodactylum tricornutum* wild‐type (WT), the transgenic control (CTR), the *RITMO1* overexpression (OE1) and knock‐out (KO2) lines under LD cycles and LL conditions.
**Fig. S12** Sequence of the pL2‐1 plasmid used to transform *Phaeodactylum tricornutum RITMO1* knock‐out KO2 to generate the complemented KO2‐C2 strain.
**Fig. S13** Selection of complemented *RITMO1* lines in *RITMO1* KO2 mutant.
**Fig. S14** Analysis of the rhythmic expression of selected genes by qRT‐PCR in *Phaeodactylum tricornutum* wild‐type (WT), *RITMO1* ectopic overexpression (OE1), *RITMO1* knock‐out KO2 and the KO2‐C2 complemented strains (*n* ≥ 6) in free running conditions.
**Fig. S15** Scheme of the validated and putative components of the diatom circadian clock system.
**Methods S1** Complementation of *RITMO1* knock‐out strain.
**Methods S2** Analysis of photosynthetic parameters.
**Methods S3** Cell cycle analysis.
**Table S1** List of the spacer sequences for gRNAs and oligonucleotides used in this work.
**Table S2** Rhythmicity parameters and growth of *Phaeodactylum tricornutum* cells grown under LD cycles of different light intensities and periods and following a shift to different free running conditions.
**Table S3** Rhythmicity parameters and growth of *Phaeodactylum tricornutum* wild‐type (WT), the transgenic control (CTR), the *RITMO1* ectopic overexpression (OE1) and knock‐out (KO1 and KO2) lines adapted to different photoperiods.
**Table S4** Rhythmicity parameters and growth of *Phaeodactylum tricornutum* wild‐type (WT), the transgenic control (CTR), *RITMO1* ectopic overexpression (OE1), the knock‐out (KO1 and KO2) lines, and the KO2 complemented line KO2‐C2 in free‐running LL conditions.
**Table S5** Analysis of rhythmicity of the *Phaeodactylum tricornutum* photosynthetic parameters in wild‐type (WT), RITMO1 ectopic overexpression (OE1), and the knock‐out (KO1 and KO2) lines in LD and LL.
Please note: Wiley is not responsible for the content or functionality of any Supporting Information supplied by the authors. Any queries (other than missing material) should be directed to the *New Phytologist* Central Office.

## Data Availability

The data that support the findings of this study are openly available in figshare at doi: 10.6084/m9.figshare.28546562.v1.
