# Supplementary material for: Circadian regulation of key physiological processes by the RITMO1 clock protein in the marine diatom Phaeodactylum tricornutum
Source: New Phytol. 2025 Apr 2;246(4):1724–39. doi: 10.1111/nph.70099 (PMC12018780; doi:10.1111/nph.70099)
Supplement: Supplementary file 1 — Fig. S1 Evolution of non‐photochemical quenching (NPQ) vs light intensity and kinetics of NPQ relaxation in darkness. Fig. S2 Characterisation of growth, cellular fluorescence and cell cycle synchronisation in Phaeodactylum tricornutum cells (n ≥ 3) entrained under 16 h : 8 h, light : dark cycles, 25 μmol photons m−2 s−1. Fig. S3 Characterisation of cellular fluorescence rhythmicity in Phaeodactylum tricornutum WT strain entrained under 16 h : 8 h, light : dark cycles, 25 μmol photons m−2 s−1 at 18°C and after the switch to LL 17 μmol photons m−2 s−1 at various temperatures. Fig. S4 Characterisation of cellular fluorescence rhythmicity of Phaeodactylum tricornutum grown under 16 h : 8 h, ligh : dark cycles, and 14°C : 18°C temperature cycles and following a shift to LL free‐running condition. Fig. S5 Analysis of RITMO1 gene modification in knock‐out (KO) lines compared to the wild‐type (WT) sequence. Fig. S6 Characterisation of cellular fluorescence rhythmicity of Phaeodactylum tricornutum wild‐type (WT), the transgenic control (CTR), the RITMO1 knock‐out (KO1 and KO2) and ectopic overexpression (OE) lines (n ≥ 3) under light : dark (LD) cycles. Fig. S7 Analysis of growth capacity in Phaeodactylum tricornutum wild‐type (WT), the transgenic control (CTR), the RITMO1 ectopic overexpression (OE1) and knock‐out (KO1 and KO2) lines. Fig. S8 Characterisation of cellular fluorescence rhythmicity in Phaeodactylum tricornutum wild‐type (WT), the transgenic control (CTR), the RITMO1 ectopic overexpression (OE1) and knock‐out (KO1 and KO2) lines entrained at 16 h : 8 h, light : dark (LD) cycles, 25 μmol photons m−2 s−1 after the switch to LL 25 μmol photons m−2 s−1. Fig. S9 Analysis of photosynthetic parameters in the Phaeodactylum tricornutum wild‐type (WT), RITMO1 ectopic overexpression (OE1) and knock‐out (KO1 and KO2) lines. Fig. S10 Characterisation of cellular fluorescence rhythmicity in Phaeodactylum tricornutum WT (Pt4) and the Aureochrome1a knock‐out (KO8 and KO9) lin [file NPH-246-1724-s001.pdf]

New Phytologist **Supporting Information**

Article title**: Circadian regulation of key physiological processes by the RITMO1 clock protein in the marine diatom *Phaeodactylum tricornutum***

Authors: Alessandro Manzotti[1,*], Raphaël Monteil[1,*] Soizic Cheminant Navarro[1], Dany Croteau[1], Lucie Charreton[1], Antoine Hoguin[1], Nils Fabian Strumpen[2], Denis Jallet[3], Fayza Daboussi[3,4], Peter G. Kroth[2], François-Yves Bouget[5], Marianne Jaubert[1], Benjamin Bailleul[1], Jean-Pierre Bouly[1,6] and Angela Falciatore[1]

Article acceptance date: 01 March 2025

[1]Laboratoire de Photobiologie et Physiologie des Plastes et des Microalgue, UMR7141, CNRS, Sorbonne Université, Institut de Biologie Physico-Chimique, 75005 Paris, France;

[2]Fachbereich Biologie, Universität Konstanz, 78457 Konstanz, Germany ;

[3]Toulouse Biotechnology Institute (TBI), Université de Toulouse, CNRS, INRAE, INSA, 31077 Toulouse, France;

[4]Toulouse White Biotechnology (TWB), INSA, 31077 Toulouse, France;

[5]Laboratoire d'Océanographie Microbienne Sorbonne Université, CNRS UMR7621, Observatoire Océanologique, 66650 Banyuls sur Mer, France;

[6]Molécules de Communication et Adaptation des Micro-Organismes, UMR 7245, F-75231 CNRS/MNHN, Paris, France.

*These authors contributed equally to this work.

Author for correspondence:

Angela Falciatore, angela.falciatore@ibpc.fr

The following Supporting Information is available for this article:

Methods S1

Complementation of *RITMO1* knock-out strain

U loop assembly: The uLoop modular cloning approach was used to generate, according to Pollak *et al.*, (2020), a vector containing the genomic *RITMO1* sequence fused to the *Venus* reporter (RITMO1g:Venus) with its endogenous promotor and terminator, and a resistance cassette to nourseothricine with *FcpB* promotor and *FcpA* terminator. RITMO1 promotor (*RITMO1p*, -824 nt upstream start codon) and RITMO1 open reading frame (*RITMO1g*, Phatr3_J44962) blocks were newly synthetized by Integrated DNA Technologies (IDT), and the RITMO1 terminator (*RITMO1t*, +539 nt downstream stop codon) block was obtained after two rounds of PCR reaction with the primer pairs TermbHLH1.F.Rv and TermbHLH1.E.Fw, and UNS1FLFw and UNSXFLRv (Table S1). Fragments were assembled by Gibson in *Sap*I-opened pL0 as pL0_AC-RITMO1p, pL0_CD-RITMO1g and pL0_EF-RITMO1t, and subsequently assembled into pL1 pCAo2 along with Venus tag pL0_DE-Venus (obtained from Pollak et al, 2020). Nourseothricine resistance (*NrsR*) cassette was generated as pL1 pCAo2-1 vector by assembling FcpB promotor pL0_AC-FcpB (*FcpBp*) (obtained from Pollak *et al.*, (2020)), and nourseothricine resistance gene (*NrsR*) pL0_CE_NAT and FcpA terminator pL0_EF-FcpAt (*FcpAt*) (obtained after two rounds of PCR with the NAT.C.Fw and NAT.E.Rv, and the FcpAt.E.Fw and FcpAt.F.Rv primer pairs, respectively, and UNS1FLFw and UNSXFLRv primers, and Gibson cloning into SapI-opened pL0) into pL1 pCAo1. RITMO1 fusion expressing vector and resistance cassette were finally assembled with pCAo3 and pCAo4 spacers in pL2 pCAe1 to obtain the final sequence that was transformed by biolistic in KO2 background. Map of this pL2 is presented in Fig. S12.

Screening Process: After transformation by biolistic method (Falciatore *et al.*, 1999), an initial screen was performed on selective plates containing 50% F2 medium supplemented with 300 µg ml^{-1} nourseothricine. A second screen was conducted on plates with 95% F2 medium, also containing 300 µg ml^{-1} nourseothricine. The presence of the transgene in the transformed cells was confirmed by PCR (primer sequences listed in Table S1). On selected positive colonies, the expression of the RITMO1g:Venus fusion protein was characterized at ZT1 and ZT11 under 12 h : 12 h, light : dark (LD) conditions by Western blot (Fig. S13). These strains were further tested for cellular fluorescence rhythmicity. One strain, KO2-C2, successfully rescued the

cellular fluorescence phenotype under continuous light LL conditions and was chosen for further gene expression analyses under LD and LL conditions via quantitative PCR (qPCR).

Immunoblot analysis: Proteins were extracted as previously described in Coesel *et al.*, (2009). Membranes were blocked with PBS-T 5% milk (w/v) and incubated overnight at 4°C with α-GFP tag Mouse McAb (Proteintech) (1:5000) antibody. Following incubation with HRP-conjugated 521 secondary antibodies (Promega), proteins were detected with ClarityMax reagents (Bio-Rad) and imaged with a ChemiDoc Touch imaging system (Biorad).

RITMO1g:Venus subcellular localization: The KO2 line complemented with the RITMO1p::RITMO1g:Venus::RITMO1t construct where grown under LD cycles and collected 1 h before the Dark period for microscopy analysis. For chlorophyll autofluorescence cells were excited at 480/20 nm and detected at 560–840 nm, and for Venus fluorescence cells were excited at 500/20 nm and detected at 535/30 nm. Hoechst 33342 (Life Technologies) was used at a final concentration of 5µg ml^{-1} to stain nuclear DNA and stained cells were visualized by illumination at 380/30 nm and detection at 450/50 nm.

Methods S2

Analysis of photosynthetic parameters

Analysis of different photosynthetic parameters were performed by using a thorough PSII fluorescence protocol on cells grown as discussed in Materials and Methods. Fluorescence induction from a saturating single turnover flash (100 µs) of blue light (450 nm) applied on dark-acclimated samples was used to calculate the functional absorption cross-section σPSII and connectivity of PSII (Gorbunov *et al.*, 2020).

The light adapted φPSII was calculated as (Fm' - Fs) / Fm', where Fm' represents the maximum fluorescence in light-adapted conditions and Fs is the steady-state fluorescence in the light. The relative electron transport rate (rETR) was then calculated as rETR = φPSII × E. The relationship between rETR and the light intensity (E) was fitted using the equation rETR = rETRm × (1 – exp (-α × E / rETRm)), where rETRm is the maximum rETR and α is the light-limited slope of the rETR vs E curve. Only rETR values from 25 and 50 µmol photons m^{-2} s^{-1} were used for the fit due to noise in the values above 600 µmol photons m^{-2} s^{-1}.

The photoprotection capacity through Nonphotochemical quenching (NPQ) was calculated as Fm/Fm’-1, where Fm’ is the light acclimated maximal fluorescence, and the light dependence of NPQ was fitted as NPQ = NPQm × (E /(E50NPQ + E)*n, where NPQm is the

(asymptotic) maximal NPQ capacity, E50NPQ is the light irradiance at half saturation of NPQ, and n is the sigmoidicity coefficient (Serôdio & Lavaud, 2011). Kinetics of NPQ relaxation were also investigated, which strictly reflects the activity of the xanthophyll cycle in *P. tricornutum* (Blommaert *et al.*, 2021). Due to time constraints imposed by the experimental design, we only measured the first 4 min of the NPQ relaxation after the transition from the highest light step (where NPQ value is NPQ800) to darkness. From there, "NPQrel." was calculated as (NPQ800 - NPQ4min) / NPQ800, where NPQ4min is the remaining NPQ after 4 min of relaxation (Fig. S1).

Methods S3

Cell cycle analysis

Cell cycle analysis was performed in cells grown under 16 h : 8 h, light : dark (LD) cycles. Around 1-2 x10^6 cells in exponential growth were collected by centrifugation every 3h for 24h. Cell pellets were fixed in cold 70% EtOH and stored for at least 24h in the dark at 4°C until processing. Fixed cells were then washed once in cold EtOH 70%, once in PBS and stained in the dark for 45 min at room temperature with DAPI (4',6-diamidino-2-phenylindole) at a final concentration of 0.5 ng ml^{-1}. After staining, cells were washed with PBS and 50,000 events per sample were analysed by flow cytometry using the MACSQuant flow cytometer. The V1-A channel (408 nm excitation, 450/50 nm detection) was used to evaluate the content of DAPI-stained DNA. 2c and 4c peaks were used to infer the fractions of cells in different cell cycle phases, using a dedicated R script (Agier & Fischer, 2016).

Table S1

(a)

Gene	Accession	Name	Strand	Sequence	3' PAM
APT	Phatr3_J6834	APTg1	Rv	AAGCGTGGAATGCCTTTGAA	GGG
		APTg2	Fw	CCTGGGTCCACCAATTGCCC	TGG
RITMO1	Phatr3_J44962	gHLH1	Rv	TCAAGATTCGCGAAAGCAGG	CGG
		gHLH2	Fw	ATGCCGAGTGGGGCTAGAAA	CGG
		gHLH3	Fw	ATACAGTACATTGGTTACGG	TGG

(b)

Gene	Accession	Name	Strand	Sequence	Analysis
RITMO1t	Phatr3_J44962	TermbHLH1.F.Rv	Rv	taatctatgtatcctggtGGTCTCtAGCGATGTCGTCATTCGTACTTCGG	Gibson assembly
		TermbHLH1.E.Fw	Fw	gtctcgtctcgtctcaGGTCTCaGCTTGAGATGCAAATTATGTCTTTCGG	Gibson assembly
UNS		UNSXFLRv	Rv	GGTGGAAGGGCTCGGAGTTGTGGTAATCTATGTATCCTGGT	Gibson assembly
		UNS1FLFw	Fw	CATTACTCGCATCCATTCTCAGGCTGTCTCGTCTCGTCTC	Gibson assembly
Nrs Nat		NAT.E.Rv	Rv	TGGTAATCTATGTATCCTGGTGGTCTCTAAGCTCAGGGGCAGGGCATGCTC	Gibson assembly
		NAT.C.Fw	Fw	AGGCTGTCTCGTCTCGTCTCAGGTCTCAAATGACCACTCTTGACGACAC	Gibson assembly
FcpA	Phatr3_J18049	FcpAt.F.Rv	Rv	TGGTAATCTATGTATCCTGGTGGTCTCTAGCGTCTAGATGAAGACGAGCTAG	Gibson assembly
		FcpAt.E.Fw	Fw	AGGCTGTCTCGTCTCGTCTCAGGTCTCAGCTTGAATTCTGAGCTACCTCGAC	Gibson assembly
RITMO1g:Venus		So26 (Venus)	Rv	AAGGGCATCGACTTCAAGGA	screening
		ARo92 (RITMO1)	Fw	AGCAACGAATGCATGCAAGG	screening

(c)

Gene	Accession	Name	Strand	Sequence	Analysis
TBP	Phatr3_J10199	TBP_Fw	Fw	ACCGGAGTCAAGAGCACACAC	RT-qPCR
		TBP_Rv	Rv	CGGAATGCGCGTATACCAGT	RT-qPCR
RPS	Phatr3_J10847	RPS_Fw	Fw	CGAAGTCAACCAGGAAACCAA	RT-qPCR
		RPS_Rv	Rv	GTGCAAGAGACCGGACATACC	RT-qPCR
RITMO1	Phatr3_J44962	RITMO1_Fw	Fw	ATTCTTGGTCCCACCCGGTA	RT-qPCR
		RITMO1_Rv	Rv	ACGCCACATTGAAAAACCGAG	RT-qPCR
		RITMO1seq_Fw	Fw	GGGGACAAGTTTGTACAAAAAAGCAGGCTCCATGAATAAGCCAGGACAGCG	sequencing
		RITMO1seq_Rv	Rv	GGGGACCACTTTGTACAAGAAAGCTGGGTCTCAGTCTTCGGGTGTCGGTTCTG	sequencing
bHLH1b	Phatr3_J44963	bHLH1b_Fw	Fw	CAAAAGCAGCCAACGACGAA	RT-qPCR
		bHLH1b_Rv	Rv	GATATGAGACCCGACCGCTG	RT-qPCR
bHLH3	Phatr3_J42586	bHLH3_Fw	Fw	CACTCTCATCATGCGGGAAT	RT-qPCR
		bHLH3_Rv	Rv	GCGCGTTGTCTTCCTCTATC	RT-qPCR
bZIP7	Phatr3_J48800	bZIP7_Fw	Fw	CCTTATTGATATTCAAGATTCCAAGG	RT-qPCR
		bZIP7_Rv	Rv	GTTTCGGAACCTGCATAGGA	RT-qPCR
Aureo1a	Phatr3_J8113	Aureo1a_Fw	Fw	ATGTCCGAACAGCAAAAGGT	RT-qPCR
		Aureo1a_Rv	Rv	CTGGAGGGATTCCAACAAGA	RT-qPCR
CPF1	Phatr3_J27429	CPF1_Fw	Fw	CCAATTGTTGACCACAAGTTGG	RT-qPCR
		CPF1_Rv	Rv	CGATTCTTTGCACTTTCTGTTAG	RT-qPCR
CYCP6	Phatr3_J6231	CYCP6_Fw	Fw	AGGTGCTTGCTGCTGTTC	RT-qPCR
		CYCP6_Rv	Rv	ACGAGGCATACTTGTGAATCC	RT-qPCR
dsCYC2	Phatr3_J34956	dsCYC2_Fw	Fw	CTATCATCGCACTCGTCATCAAC	RT-qPCR
		dsCYC2_Rv	Rv	TGTCCACCAAAGCCTCCAAAC	RT-qPCR
CYCB1	Phatr3_J46095	CYCB1_Fw	Fw	TCCTGGTCCGCTACTTGAAAG	RT-qPCR
		CYCB1_Rv	Rv	GCTGGCTGGGAAGATAACGC	RT-qPCR
FtsZ	Phatr3_J42361	FtsZ_Fw	Fw	GTGATTTTCGGGGCTTTGGT	RT-qPCR
		FtsZ_Rv	Rv	TTCATTTCCTGTTCGCGTCC	RT-qPCR
POR2	Phatr3_J13001	POR2_Fw	Fw	CCTGGTTGCATTGCCGAATC	RT-qPCR
		POR2_Rv	Rv	TCTCCAACGTATCCTCCCGT	RT-qPCR
DPH	Phatr3_J54330	DPH1_Fw	Fw	GACATCGGGCATGTGATAGT	RT-qPCR
		DPH1_Rv	Rv	GCAATAGAGGTCCTCACAGCA	RT-qPCR

Table S1: List of the spacer sequences for gRNAs and oligonucleotides used in this work.

(a) List of the spacer sequences for gRNAs used for CRISPR-Cas9 mutagenesis of the *RITMO1* gene. Target gene, gRNA name, target strand, spacer sequence for gRNA and the associated protospacer adjacent motif (PAM) are reported. (b) List of the primers used for Gibson assembly of RITMO1t uLoop block and primers used for the screening of KO2 complemented strains (Method S3). (c) List of the oligonucleotides used for the sequencing of *RITMO1* WT and KO sequences and for RT-qPCR. Target gene, ID number in the Diatomicsbase (https://www.diatomicsbase.bio.ens.psl.eu/) (Villar *et al.*, 2025), pairing strand and sequence of the oligonucleotides used in this work. Sequencing primers for *RITMO1* are allele specific.

Table S2

Condition	16h:8h, LD 25 µmol m^{-2} s^{-1}		DD		LL 5 µmol m^{-2} s^{-1}		LL 17 µmol m^{-2} s^{-1}		LL 25 µmol m^{-2} s^{-1}		16h:8h, LD 75 µmol m^{-2} s^{-1}		LL 50 µmol m^{-2} s^{-1}		LL 75 µmol m^{-2} s^{-1}		6h:6h, LD 25 µmol m^{-2} s^{-1}	
rep. number	3		4		3		10		7		4		4		8		4	
ERP rhythmic lines	3/3		0/4		0/3		9/10		7/7		4/4		4/4		6/8		4/4	
FFT-NLLS algorythm	mean	SD	mean	SD	mean	SD	mean	SD	mean	SD	mean	SD	mean	SD	mean	SD	mean	SD
Period	23,98	0,23	NA	NA	NA	NA	27,77	1,19	28,54	1,64	23,84	0,99	27,84	3,96	25,98	2,42	11,78	0,16
Phase	13,28	0,54	NA	NA	NA	NA	12,7	1,04	11,62	0,79	14,33	1,45	13,01	2,3	14,02	2,07	14,4	1,5
Amplitude	1,41	0,04	NA	NA	NA	NA	0,63	0,19	0,43	0,03	0,12	0,01	0,28	0,03	0,29	0,05	0,9	0,15
RAE	0,16	0,03	NA	NA	NA	NA	0,43	0,11	0,42	0,1	0,25	0,07	0,46	0,11	0,56	0,11	0,64	0,08
div. per day	0,97	0,03	NA	NA	0,2	0,07	1,07	0,19	1,09	0,06	1,49	0,09	1,24	0,05	1,4	0,03	0,49	0,14

Free running Temperature	14°C		16°C		18°C		20°C		22°C		LD + T entr. 18°C-14°C	
rep. number	8		3		10		3		8		6	
ERP rhythmic lines	8/8		3/3		9/10		3/3		3/8		6/6	
FFT-NLLS algorithm	mean	SD	mean	SD	mean	SD	mean	SD	mean	SD	mean	SD
Period	24,7	1,85	23,84	2,77	27,77	1,19	27,28	0,93	25,67	4,37	25,64	1,45
Phase	14,34	3,04	9,37	5,72	12,70	1,04	11,87	1,43	16,62	2,93	10,04	3,48
Amplitude	0,58	0,10	0,84	0,27	0,63	0,19	0,72	0,19	0,88	0,29	0,79	0,14
RAE	0,5	0,14	0,46	0,23	0,43	0,11	0,53	0,20	0,66	0,05	0,44	0,07
div. per day	0,97	0,10	1,22	0,03	1,18	0,03	1,33	0,06	1,05	0,13	1,14	0,06

Table S2: Rhythmicity parameters and growth of *P. tricornutum* cells grown under light : dark (LD) cycles of different light intensities and periods and following a shift to different free running conditions.

Data in the upper table refer to wild-type (WT) cells entrained under 16 h : 8 h, LD 25 µmol photons m^{-2} s^{-1} at 18°C and subsequently exposed to different free-run conditions over four subjective days (DD, LL 5 µmol photons m^{-2} s^{-1}, LL 17 µmol photons m^{-2} s^{-1}, LL 25 µmol photons m^{-2} s^{-1}) or to cells entrained under 16 h : 8 h LD 75 µmol photons m^{-2} s^{-1} and after transition to LL of 50 and 75 µmol photons m^{-2} s^{-1}. Last column refers to data from cells entrained in 12 h : 12h LD 25 µmol photons m^{-2} s^{-1} and subsequently exposed to 6 h: 6 h, LD 25 µmol photons m^{-2} s^{-1}.
Data in the lower table refer to WT cells entrained under 16 h : 8 h, LD 25 µmol photons m^{-2} s^{-1} at 18°C and subsequently exposed to LL 17 µmol photons m^{-2} s^{-1} at different temperature ranges: 14°C, 16°C (with a temperature shift at the last light/dark transition) and 20°C and 22°C (with a temperature shift at the last dark/light transition). The last column refers to WT cells

entrained under 16 h : 8 h, LD light cycles 25 µmol photons m^{-2} s^{-1} and 8°C-14°C temperature cycles and subsequently exposed to LL 17 µmol photons m^{-2} s^{-1}. SD, Standard Deviation.
Rep. number indicates the number of biological replicas used for the analysis. EPR indicates the lines that passed the Enright Periodogram (EPR) algorithm test for each condition and that are used for the FFT-NLLS (Fast Fourier Transform Non-Linear Least Square Algorithm) rhythmicity test below. The FFT-NLLS algorithm was used to get the period, phase, amplitude, the Relative Amplitude of Error (RAE), representing the amplitude of the error between the fit used by the algorithm and the data divided by the amplitude of the oscillations. The cell division/day was calculated in cells in exponential phase over the course of four days (n=3) for all lines (including the lines found non rhythmic by EPR analysis). SD, Standard Deviation. NA, Not Applicable.

Table S3

16h:8h, LD	WT		CTR			OE1			KO1			KO2		
rep. Number	3		3			3			3			3		
ERP rhythmic lines	3/3		3/3			3/3			3/3			3/3		
FFT-NLLS algorythm	mean	SD	mean	SD	p. value	mean	SD	p. value	mean	SD	p. value	mean	SD	p. value
Period	23,98	0,23	24,25	0,26	n. s.	23,09	0,19	0,0067	24,00	0,15	n. s.	24,21	0,11	n. s.
Phase	13,28	0,54	12,16	0,52	n. s.	14,38	0,60	n. s.	13,03	0,37	n. s.	12,44	0,41	n. s.
Amplitude	1,41	0,04	1,31	0,28	n. s.	1,06	0,05	0,0007	1,32	0,17	n. s.	1,34	0,10	n. s.
RAE	0,16	0,01	0,16	0,04	n. s.	0,33	0,05	0,0095	0,19	0,03	n. s.	0,20	0,02	n. s.
div. per day	1,15	0,04	1,09	0,03	n. s.	1,06	0,16	n. s.	0,91	0,29	n. s.	0,90	0,27	n. s.

12h:12h, LD	WT		CTR			OE1			KO1			KO2		
rep. Number	4		3			3			3			3		
ERP rhythmic lines	4/4		3/3			3/3			3/3			3/3		
FFT-NLLS algorythm	mean	SD	mean	SD	p. value	mean	SD	p. value	mean	SD	p. value	mean	SD	p. value
Period	24,47	0,38	23,84	0,28	n. s.	24,44	0,58	n. s.	24,13	0,27	n. s.	25,08	1,04	n. s.
Phase	10,65	1,00	11,85	0,14	n. s.	10,88	1,23	n. s.	11,28	0,90	n. s.	10,23	1,52	n. s.
Amplitude	1,12	0,22	1,08	0,13	n. s.	0,91	0,13	n. s.	1,05	0,09	n. s.	1,15	0,06	n. s.
RAE	0,15	0,03	0,12	0,01	n. s.	0,14	0,02	n. s.	0,15	0,05	n. s.	0,19	0,03	n. s.
div. per day	0,81	0,08	0,72	0,11	n. s.	0,70	0,04	n. s.	0,65	0,13	n. s.	0,75	0,06	n. s.

8h:16h, LD	WT		CTR			OE1			KO1			KO2		
rep. Number	4		3			3			3			3		
ERP rhythmic lines	4/4		3/3			3/3			3/3			3/3		
FFT-NLLS algorythm	mean	SD	mean	SD	p. value	mean	SD	p. value	mean	SD	p. value	mean	SD	p. value
Period	23,63	0,16	23,83	0,14	n. s.	24,36	0,55	0,0486	24,05	0,46	n. s.	24,68	0,99	n. s.
Phase	9,78	0,13	9,84	0,44	n. s.	9,00	0,70	n. s.	9,73	0,23	n. s.	8,58	1,70	n. s.
Amplitude	0,27	0,03	0,24	0,03	n. s.	0,22	0,02	0,0332	0,25	0,03	n. s.	0,22	0,04	n. s.
RAE	0,19	0,03	0,23	0,09	n. s.	0,19	0,06	n. s.	0,21	0,07	n. s.	0,27	0,01	0,0090
div. per day	0,79	0,07	0,78	0,02	n. s.	0,85	0,11	n. s.	0,76	0,07	n. s.	0,76	0,05	n. s.

Table S3: Rhythmicity parameters and growth of *P. tricornutum* wild-type (WT), the transgenic control (CTR), the *RITMO1* ectopic overexpression (OE1) and knock-out (KO1 and KO2) lines adapted to different photoperiods.

The experiments were realised in cells adapted to 25 µmol photons m^{-2} s^{-1} at different photoperiods, as represented in Fig. 2: 16 h : 8 h, LD; 12 h : 12 h, LD; 8 h :16 h, LD. Rep. number indicates the number of biological replica used for the analyses. EPR indicates the lines that passed the Enright Periodogram (EPR) algorithm test for each condition and that are used for the FFT-NLLS (Fast Fourier Transform Non-Linear Least Square Algorithm) rhythmicity test below. The FFT-NLLS was used to get the period, phase, amplitude, the Relative Amplitude of Error (RAE). The cell division/day was calculated in cells in exponential phase over the course of four days (n=3) for all lines. Statistical differences were examined using unpaired Student's t-test with the WT as reference sample. Data for 16 h : 8 h, LD are the same than those shown in Table S1, here reproposed to facilitate the comparisons between the different conditions. SD, Standard Deviation; n.s., non-significant.

Table S4

Strain	WT		CTR			OE1			KO1			KO2			KO2-C2		
rep. number	17		12			7			17			15			8		
ERP rhythmic lines	17/17		12/12			5/7			11/17			9/15			7/8		
FFT-NLLS algorythm	mean	SD	mean	SD	p. value	mean	SD	p. value	mean	SD	p. value	mean	SD	p. value	mean	SD	p. value
Period	28,58	2,27	28,80	1,53	n.s.	28,12	7,57	n.s.	25,83	2,69	0,0072	24,93	6,72	0,0008	26,89	3,13	n.s.
Phase	12,16	1,36	12,62	1,32	n.s.	12,48	2,38	n.s.	14,05	2,19	0,0091	13,73	2,58	n.s.	13,25	4,93	n.s.
Amplitude	0,96	0,45	0,97	0,50	n.s.	0,62	0,39	n.s.	0,89	0,42	n.s.	0,87	0,54	n.s.	0,99	0,29	n.s.
RAE	0,39	0,13	0,37	0,11	0,6739	0,69	0,11	0,0001	0,58	0,15	0,0015	0,63	0,19	0,0008	0,45	0,12	n.s.
div. per day	1,18	0,02	1,26	0,07	n.s.	1,23	0,06	n.s.	1,18	0,03	n.s.	1,23	0,02	n.s.	1,27	0,04	n.s.

Table S4: Rhythmicity parameters and growth of *P. tricornutum* wild-type (WT), the transgenic control (CTR), *RITMO1* ectopic overexpression (OE1), the knock-out (KO1 and KO2) lines, and the KO2 complemented line KO2-C2 in free-running LL conditions.

The experiments were realised with cells adapted to 16 h : 8 h, LD 25 µmol photons m^{-2} s^{-1} and transferred to LL of 17 µmol photons m^{-2} s^{-1} (Fig. 3 and Fig. 7). Rep. number indicates the number of biological replicas used for the analysis. EPR indicates the lines that passed the Enright Periodogram (EPR) algorithm test for each condition and that are used for the FFT-NLLS (Fast Fourier Transform Non-Linear Least Square Algorithm) rhythmicity test below. The FFT-NLLS was used to get the period, phase, amplitude, the Relative Amplitude of Error (RAE). The cell division/day was calculated in cells in exponential phase over the course of four days (n=3) for all lines. Statistical differences were examined using unpaired Student's t-test with the WT as reference sample. SD, Standard Deviation; n.s., non-significant.

Table S5

Parameter	16 h : 8 h, LD			LL			
	Rhythmicity p. value	Phase	Amp.	Rhythmicity p. value	Period	Phase	Amp.
FvFm KO1	5,13E-05 ***	8	0,041	1,00E+00	28	22	0,008
FvFm KO2	1,05E-05 ***	8	0,043	1,00E+00	24	16	0,004
FvFm OE1	4,17E-08 ***	8	0,04	1,66E-01	24	16	0,007
FvFm WT	1,31E-06 ***	8	0,044	9,00E-07 ***	24	16	0,013
E50NPQ KO1	1,35E-02 *	20	31,9	1,00E+00	28	4	9,13
E50NPQ KO2	3,25E-04 ***	18	36,2	1,00E+00	20	18	6,44
E50NPQ OE1	1,05E-05 ***	18	40	1,00E+00	20	22	8,74
E50NPQ WT	5,69E-06 ***	18	42,3	6,08E-05 ***	28	2	28,2
NPQrel KO1	2,76E-04 ***	20	20,4	1,00E+00	28	6	2,9
NPQrel KO2	1,01E-03 **	18	23,7	9,47E-02	28	6	2,66
NPQrel OE1	4,18E-08 ***	16	22,6	1,00E+00	28	22	1,85
NPQrel WT	9,77E-05 ***	18	27,4	2,95E-07 ***	24	8	11,7
σPSII KO1	4,91E-07 ***	22	16,8	1,00E+00	28	4	1,84
σPSII KO2	4,26E-05 ***	22	21,2	1,00E+00	20	6	3,82
σPSII OE1	5,07E-06 ***	20	14	1,66E-01	24	4	3,18
σPSII WT	5,69E-06 ***	22	18	8,66E-03 **	24	4	5,37
rETRm KO1	1,57E-06 ***	8	7,76	1,00E+00	28	6	0,803
rETRm KO2	4,18E-06 ***	8	7,52	1,00E+00	28	10	1,62
rETRm OE1	2,13E-07 ***	8	6,65	1,00E+00	28	14	1,19
rETRm WT	9,32E-09 ***	8	7,96	1,00E+00	28	10	0,818
alpha KO1	2,22E-02 *	8	0,044	4,46E-01	28	22	0,036
alpha KO2	1,04E-02 *	12	0,047	1,00E+00	28	24	0,032
alpha OE1	2,00E-02 *	12	0,053	9,70E-01	28	26	0,012
alpha WT	6,08E-02	8	0,043	1,00E+00	20	14	0,006
NPQm KO1	2,58E-06 ***	6	0,162	1,00E+00	28	8	0,078
NPQm KO2	9,16E-06 ***	6	0,157	1,00E+00	28	8	0,07
NPQm OE1	1,11E-06 ***	4	0,144	9,64E-02	28	12	0,062
NPQm WT	2,77E-05 ***	6	0,14	1,00E+00	28	10	0,047

Table S5: Analysis of rhythmicity of the *P. tricornutum* photosynthetic parameters in wild-type (WT), *RITMO1* ectopic overexpression (OE1), and the knock-out (KO1 and KO2) lines in LD and LL.

Cells were grown under 16 h : 8 h, LD (50 µmol photons m^{-2} s^{-1}) cycles and in LL (30 µmol photons m^{-2} s^{-1}) as described in Fig. 5. From top to bottom: maximum dark-acclimated quantum yield of PSII (Fv/Fm), E50NPQ, e.g., light intensity where 50% of the maximum NPQ is reached, NPQ relaxation, *e.g.*, percentage of NPQ at maximum light intensity relaxed after 4 minutes in the dark effective absorption, cross-section of PSII (σPSII), maximum of the relative electron transfer rate (rETRm), initial light limited slope of rETR (alpha) and maximal NPQ measured (NPQm). JTK cycle analysis was performed on the dataset obtained from four biological replicas for each strain with the replica option selected (n=4). In LD, due to the reduced period of sampling a 24 h period was enforced. In LL, the period was free to vary between 20 and 28 h. Rhythmicity p. value indicates the p. value of rhythmicity provided by

JTK cycle algorithm after a Benjamini-Hochberg correction. Phase design the acrophase relative to the first LD or theoric LD transition. Amp. designs the amplitude of the variation. Rhythmicity p. value significance is noted with stars, * = $p<0.05$; ** = $p<0.01$;*** = $p<0.001$.

Fig. S1

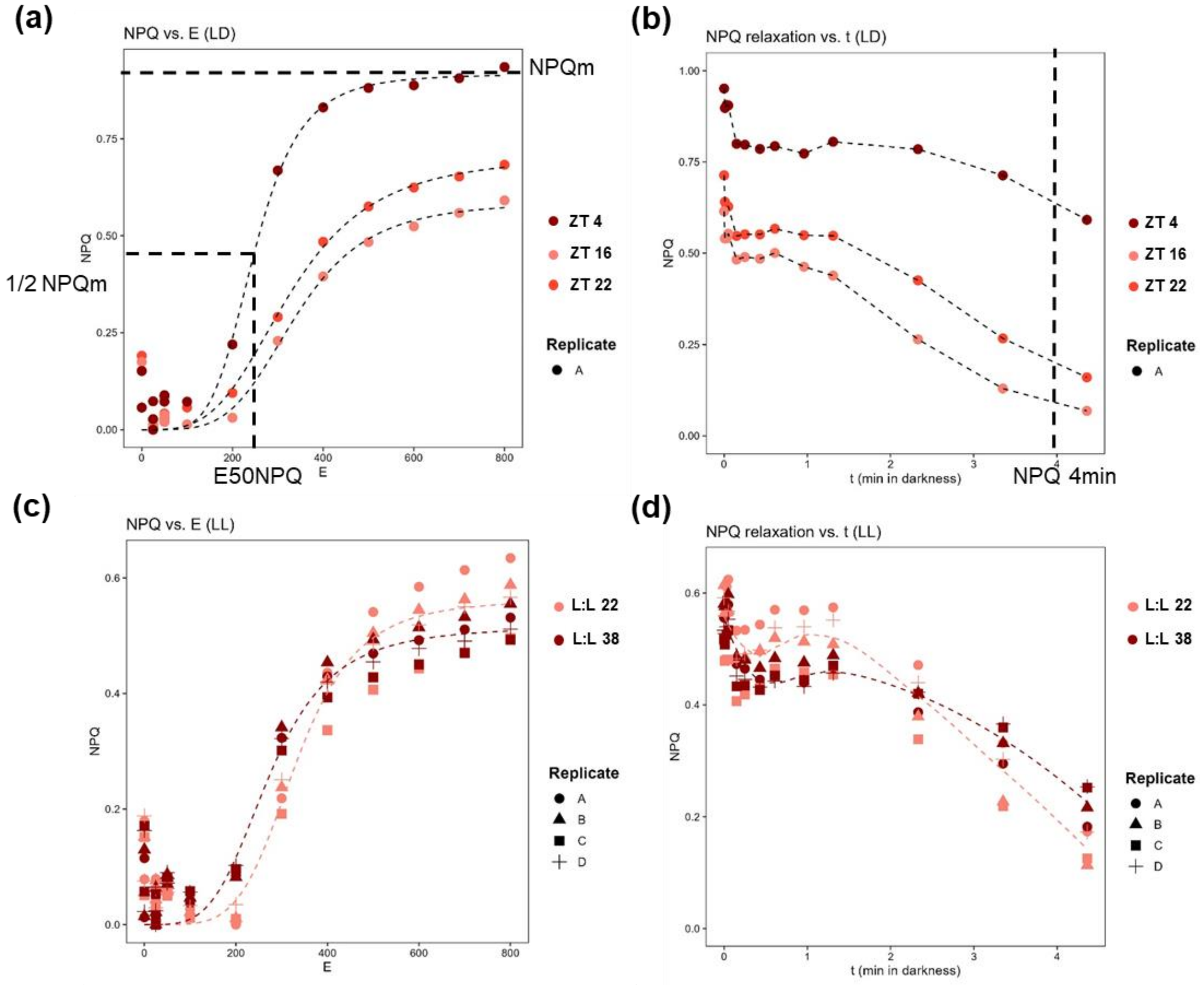

Fig. S1: Evolution of non-photochemical quenching (NPQ) versus light intensity and kinetics of NPQ relaxation in darkness.

(a, c) Relation between non-photochemical quenching (NPQ) and light intensity (E) for *P. tricornutum* wild-type (WT) strain under 16 h : 8 h, LD 25 µmol photons m^{-2} s^{-1} (a) and transferred to LL 17 µmol photons m^{-2} s^{-1} (c) at various sampling time. Light intensity for which 50% of maximum NPQ (E50NPQ) and maximum NPQ (NPQm) are indicated by thick dashed lines. (b, d) Relaxation of the NPQ in the dark after NPQm induction for WT strain in both LD (b) and LL (d) at various sampling times. In LD conditions, data are shown only for replicate A at ZT4, ZT16, and ZT22 to emphasize the maximum contrast in parameter values. In LL conditions, all four replicates are presented around LL 18 and LL 22 to capture both maximal contrast and replicate variability. Light dashed lines represent the fit for this parameter (Materials and Methods). The NPQ 4 min used to calculate NPQrel (percentage of maximal NPQ monitored relaxed after 4 min in dark) is indicated by thick dashed lines.

Fig. S2

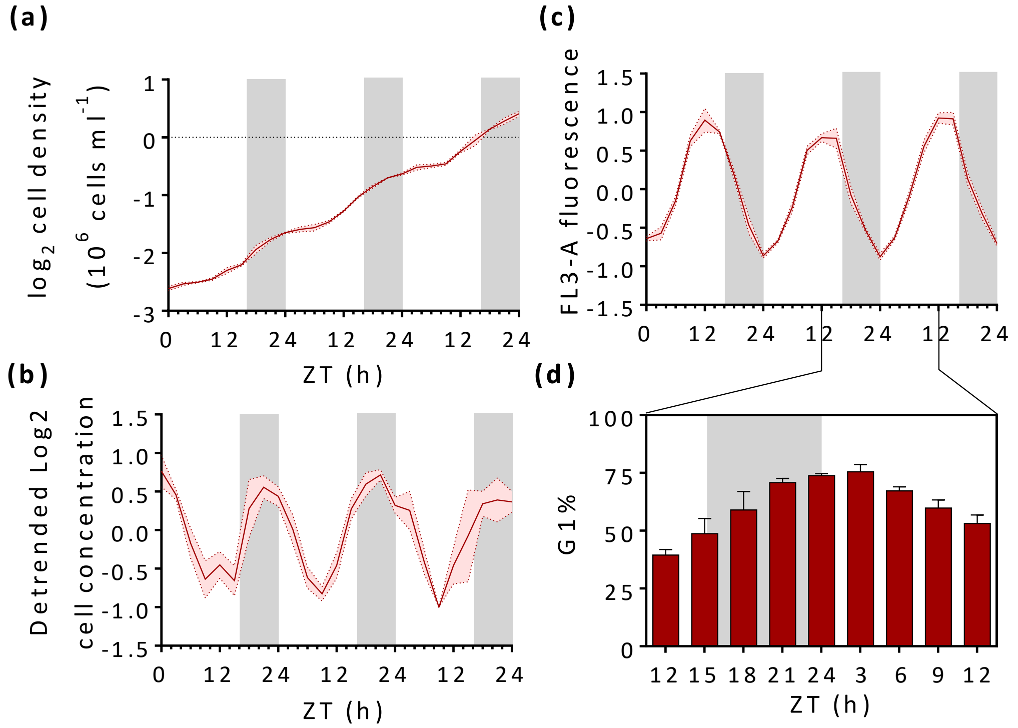

Fig. S2: Characterisation of growth (a, b), cellular fluorescence (c) and cell cycle synchronization (d) in *P. tricornutum* cells (n>=3) entrained under 16 h : 8 h, LD cycles, 25 µmol photons m^{-2} s^{-1}.

Samples were collected every 3 h for the analyses. Cell concentration plotted as 10^6 cells ml^{-1} (a). Log2 of the cell concentration presented in panel a after a baseline detrending to highlight diel variations (b). Cellular fluorescence (FL3-A parameter) profile of the data reported in panel a and b (c). Solid lines represent the average, coloured envelopes represent Standard Deviation, SD. Percentage of cells in G1 phase in the highlighted time window (d). Cell phase was determined by flow cytometry after DAPI staining of the cellular DNA content (V1-A parameter) as described in Method S3. Red bars represent the average and error bar the SD. White and grey regions represent light and dark periods.

Fig. S3

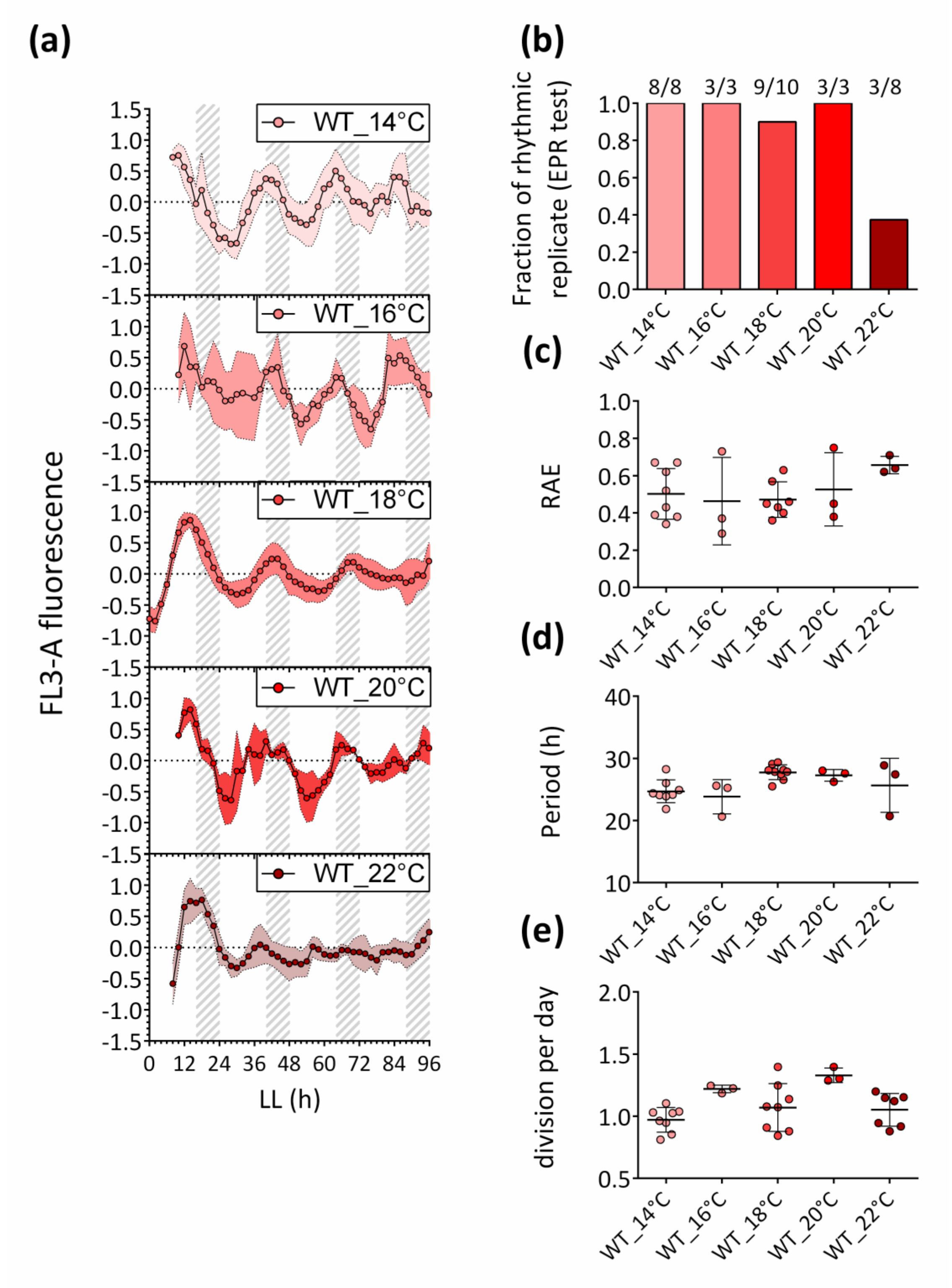

Fig. S3: Characterization of cellular fluorescence rhythmicity in *P. tricornutum* wild-type (WT) strain entrained under 16 h : 8 h, LD cyles, 25 µmol photons m^{-2} s^{-1} at 18°C and after the switch to LL 17 µmol photons m^{-2} s^{-1} at various temperatures.

Shift to lower temperature were done at the beginning of the last night (ZT 16) and shift to upper temperature at the end of the last night (ZT 24). Normalised and baseline detrended circadian cellular FL3-A fluorescence profiles in WT at 14°C, 16°C, 18°C, 20°C and 22°C (a)

(n>=3). Dots represent mean FL3-A fluorescence values, coloured envelopes represent Standard Deviation, SD. Grey dashed regions represent subjective nights in free-run conditions. (b) Fraction of replicates that passes the Enright Periodogram (EPR) algorithm test for all conditions. (c) Relative amplitude of Error (RAE) of the FFT-NLLS (Fast Fourier Transform Non-Linear Least Square Algorithm) method fit for the lines found rhythmic with EPR test. (d) Period estimation obtained with FFT-NLLS method. (e) Division per day along the experiment (n>=3). Dots represent mean values, error bars represent SD.

Fig. S4

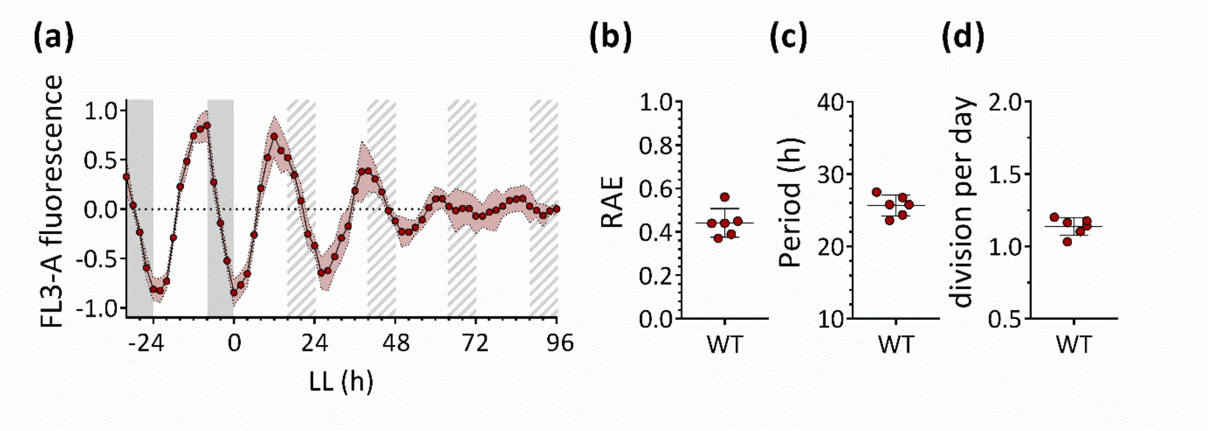

Fig. S4: Characterisation of cellular fluorescence rhythmicity of *P. tricornutum* grown under 16 h : 8 h, LD cycles and 14 °C : 18°C temperature cycles and following a shift to free-running condition.

(a) Fluorescence profile (FL3-A parameter) of wild-type (WT) cells adapted to 16 h : 8 h, LD cycles (25 µmol photons m^{-2} s^{-1}) with 18°C during the light and 14°C during the dark, and subsequently exposed to LL 17 µmol photons m^{-2} s^{-1} and 18°C (n=6). (b) Relative amplitude of Error (RAE) of the FFT-NLLS (Fast Fourier Transform Non-Linear Least Square Algorithm) method fit; (c) Period estimation obtained with FFT-NLLS method and calculated under free running. (d) Division per day along the experiment (n=6). Fluorescence profiles were baseline detrended and then normalized between 1 and -1 for graphic representation. Colored envelopes represent standard deviations. White and grey regions represent light and dark periods, grey dashed regions represent subjective nights in LL. The time is counted starting from the end of the last LD day.

Fig. S5

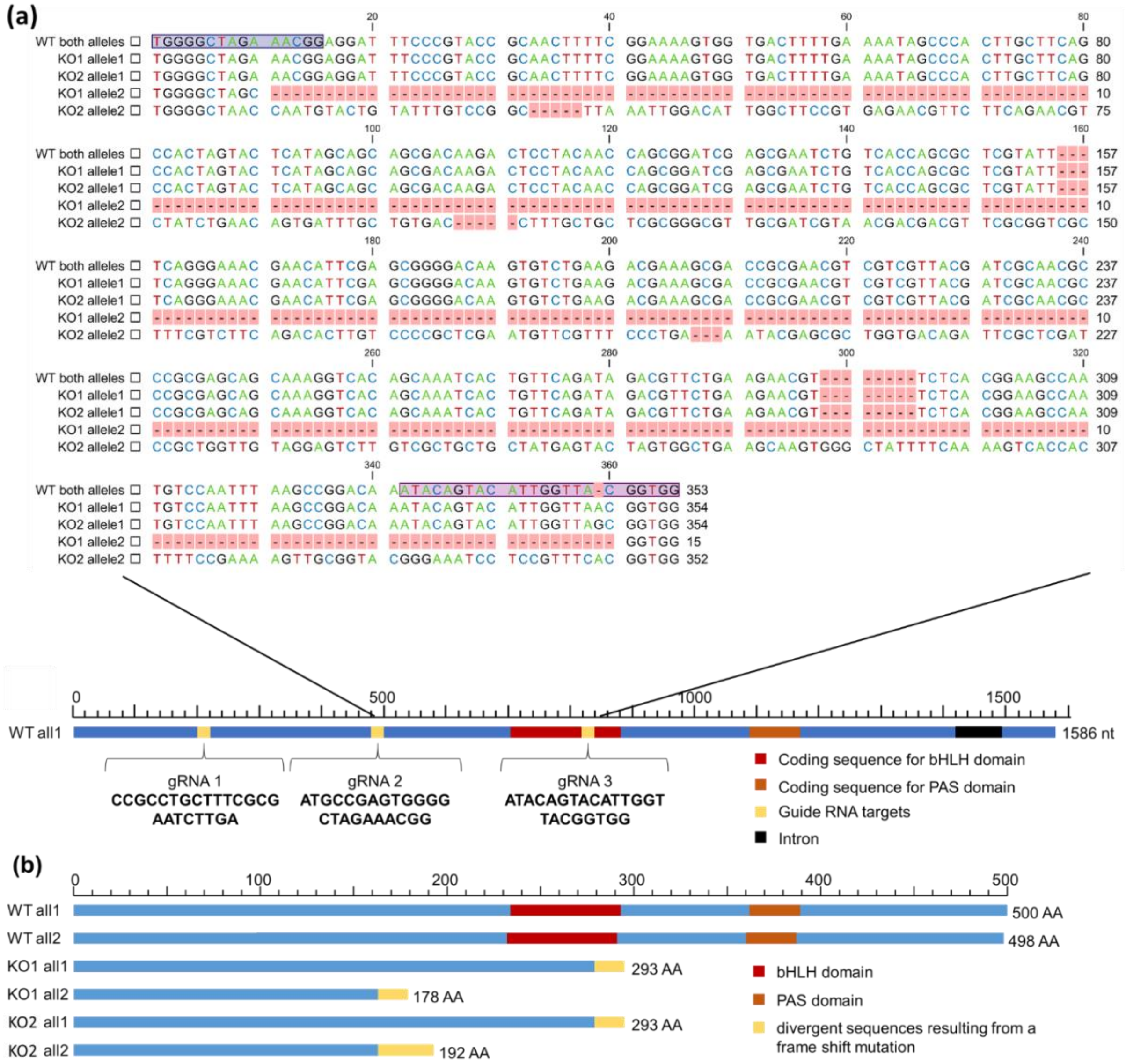

Fig. S5: Analysis of *RITMO1* gene modification in knock-out (KO) lines compared to the wild-type (WT) sequence.

(a) Schematic representation of the *P. tricornutum RITMO1* locus on chromosome 5 (Phatr3_J44962 in https://www.diatomicsbase.bio.ens.psl.eu/). The intron is represented by a black box. Red and orange boxes indicate the relative position of the coding sequence for bHLH and PAS domains, respectively. Yellow boxes indicate the location of the target sequences (underlined) of the three gRNAs used for *RITMO1* CRISPR-Cas9 mutagenesis. The alignment of the regions containing the mutations in both alleles of *RITMO1* KOs compared to the WT sequence is shown at the top. (b) Schematic representation of the RITMO1 protein encoded by the two alleles of the *P. tricornutum* WT strain and the two KO mutants used in this study and identified by Sanger sequencing. Red and orange boxes indicate the relative positions of the bHLH and PAS domains, respectively, as predicted by InterProScan. Yellow boxes highlight sequence divergence from WT resulting from a frame shift mutation.

Fig. S6

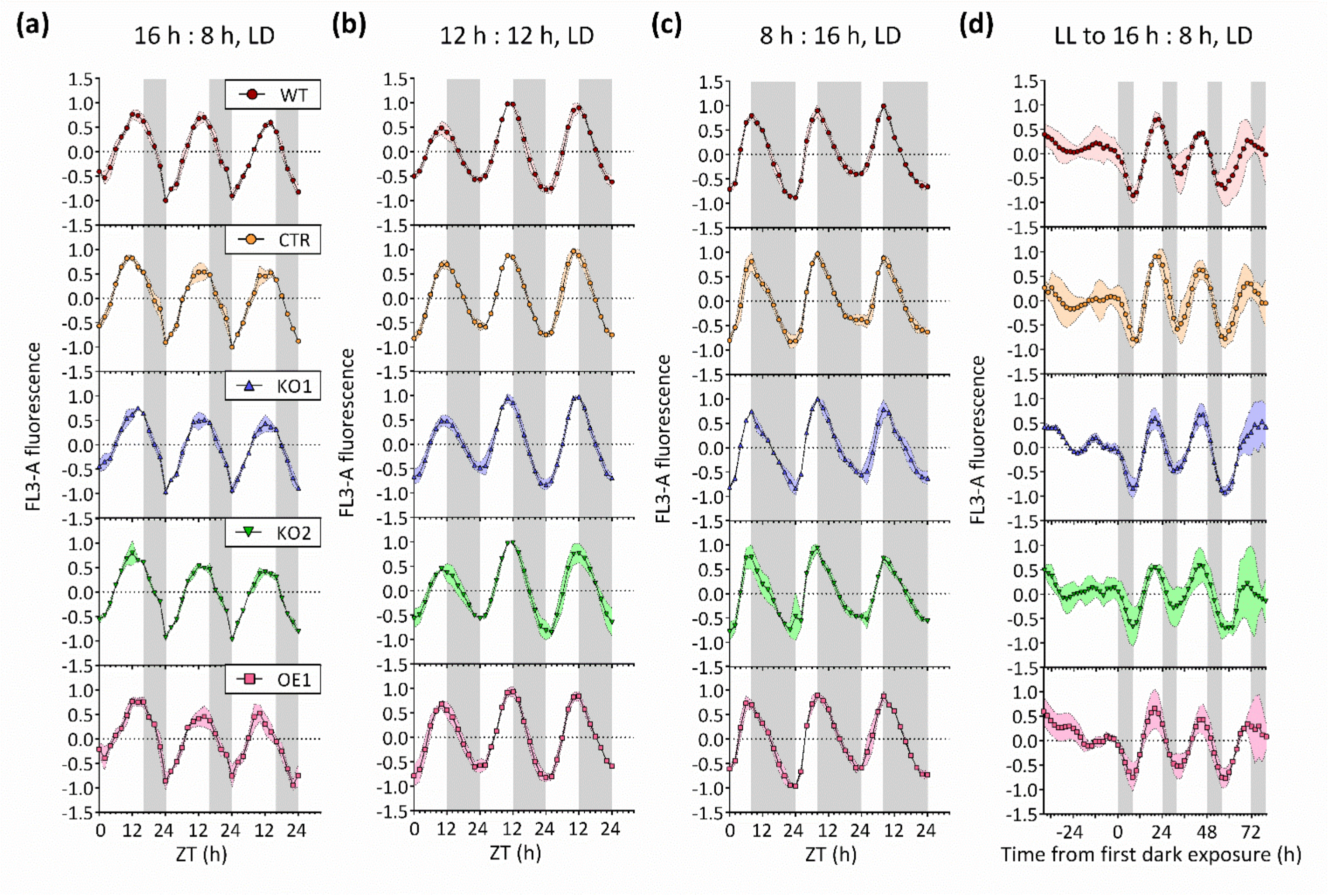

Fig. S6: Characterization of cellular fluorescence rhythmicity of *P. tricornutum* wild-type (WT), the transgenic control (CTR), the *RITMO1* knock-out (KO1 and KO2) and ectopic overexpression (OE) lines (n>=3) under LD cycles. In (a-c), the experimental conditions and data are the same as in Fig. 2, but are represented as individual data for each strain to improve visualization of results. (d), re-entrainment experiments as for Fig. 4, with the same criteria.

Fig. S7

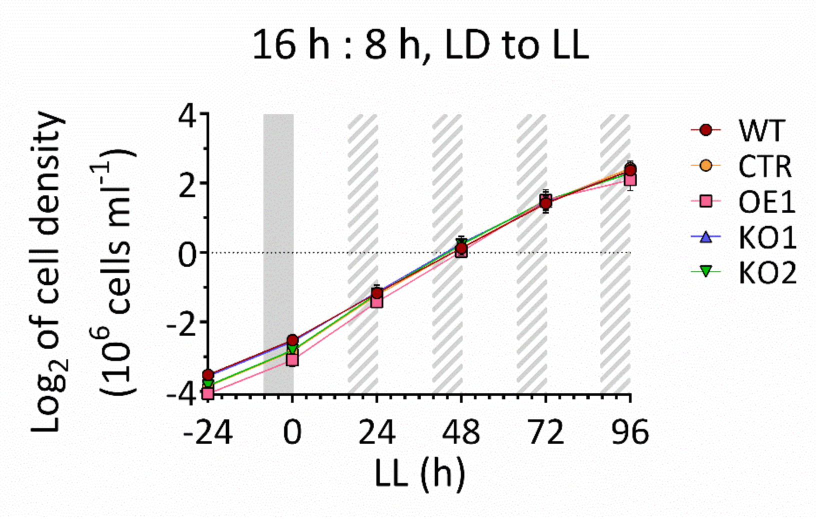

Fig. S7: Analysis of growth capacity in *P. tricornutum* wild-type (WT), the transgenic control (CTR), the *RITMO1* ectopic overexpression (OE1) and knock-out (KO1 and KO2) lines.

Growth curve of *P. tricornutum* WT, CTR, *RITMO1* KO1, KO2 and OE1 cells (n=3) under 16 h : 8 h, light : dark (LD) cycles, 25 µmol photons m^{-2} s^{-1} and transferred to LL 17 µmol photons m^{-2} s^{-1}. Dots represent mean value of the log_2 of the cell density, error bar indicates Standard Deviations, SD.

Fig. S8

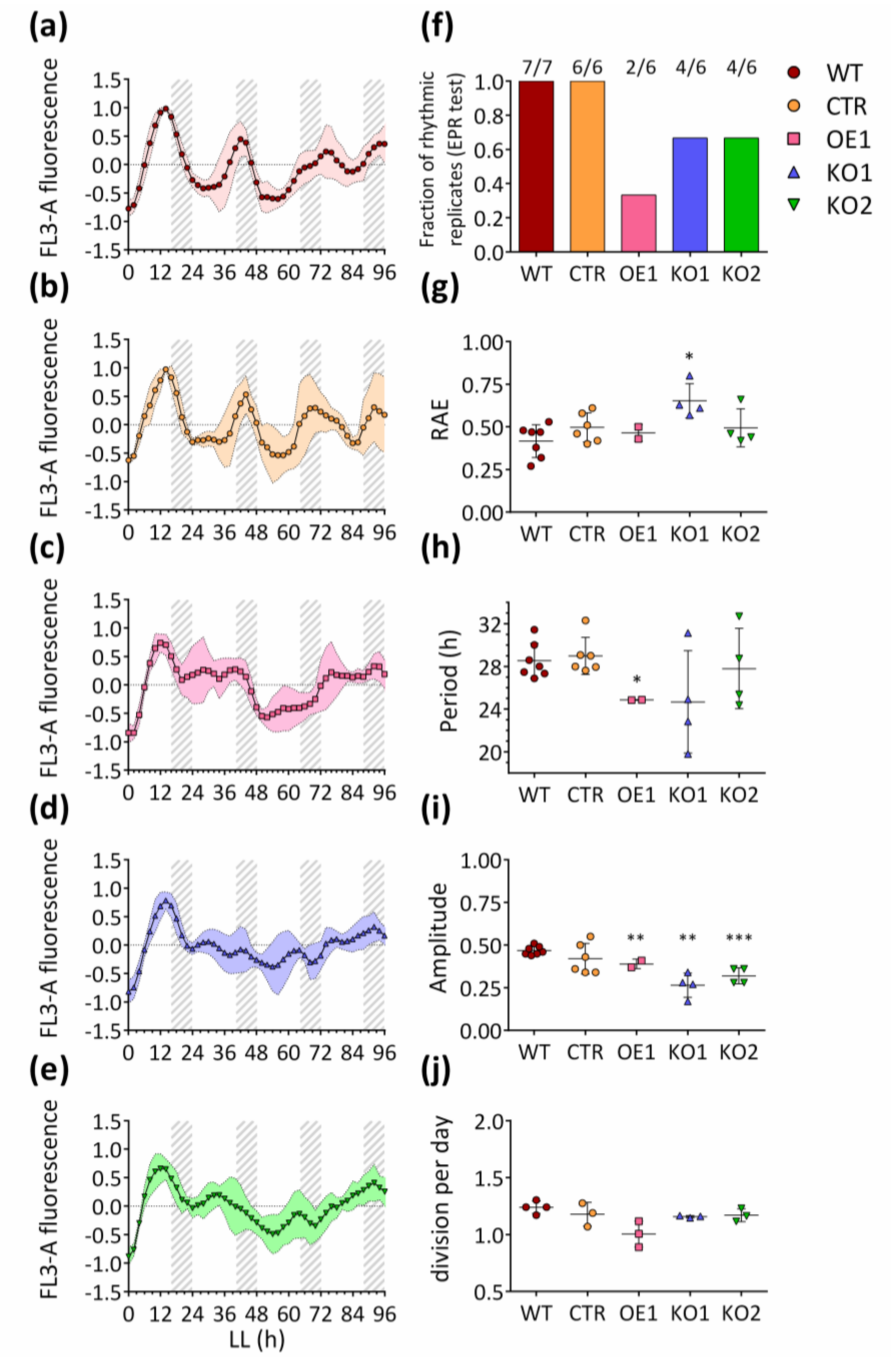

Fig. S8: Characterization of cellular fluorescence rhythmicity in *P. tricornutum* wild-type (WT), the transgenic control (CTR), the *RITMO1* ectopic overexpression (OE1) and knock-out (KO1 and KO2) lines entrained at 16 h : 8 h, light : dark (LD) cycles, 25 µmol photons m^{-2} s^{-1} after the switch to LL 25 µmol photons m^{-2} s^{-1}.

Normalised and baseline detrended circadian cellular FL3-A fluorescence profiles in WT (a), CTR (b), OE1 (c), KO1 (d) and KO2 (e) (n>=6). Dots represent mean FL3-A fluorescence values, coloured envelopes represent Standard Deviation (SD). Gray dashed regions represent subjective nights in free-run conditions. (f) Fraction of replicates that passes the Enright Periodogram (EPR) algorithm test for all strains. (g) Relative amplitude of Error (RAE) of the FFT-NLLS (Fast Fourier Transform Non-Linear Least Square Algorithm) method fit for the

lines found rhythmic with EPR test. (h) Period estimation obtained with FFT-NLLS method. (i) Relative amplitude of Error of the FFT-NLLS method fit vs the predicted period for the lines found rhythmic with EPR test. (j) Division per day along the experiment (n>=3).

Dots represent mean values, error bars represent SD. Statistical differences were examined using unpaired Student's t-test with the WT as reference sample (* = $p<0.05$; ** = $p<0.01$;*** = $p<0.001$).

Fig. S9

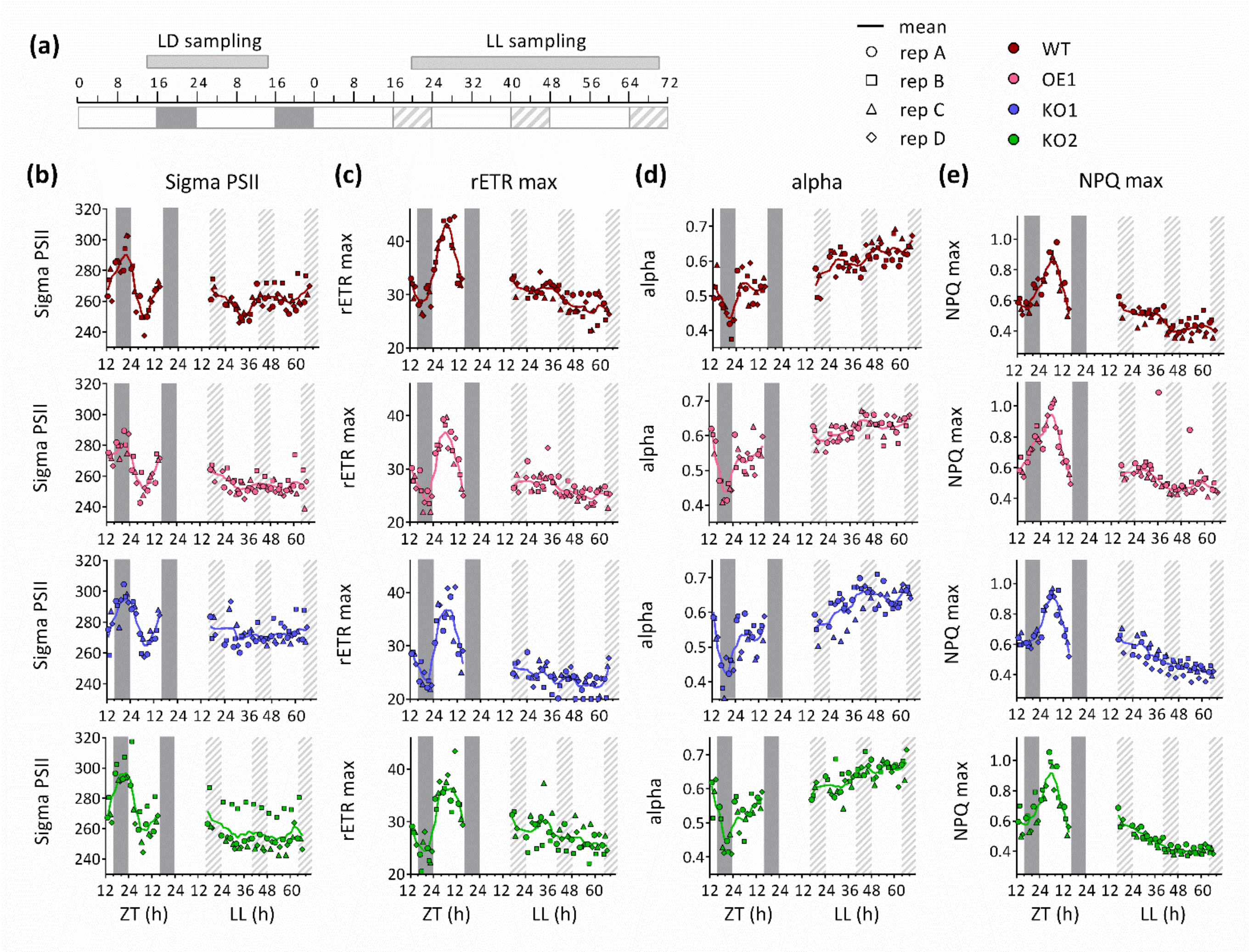

Fig. S9: Analysis of photosynthetic parameters in the *P. tricornutum* wild-type (WT), *RITMO1* ectopic overexpression (OE1) and knock-out (KO1 and KO2) lines.

(a) Schematic representation of sampling times for the analysis from WT, *RITMO1* KO1, KO2 and OE1 cells grown under 16 h : 8 h, LD 50 µmol photons m^{-2} s^{-1} and released to LL 30 µmol photons m^{-2} s^{-1}. Samples were collected continuously and measured one by one in the following order WT, KO1, KO2, OE1 from replicate A to replicate D, with 15 min interval due to the measurement. The measure of all strains and all replicates takes 4 h, measurement were performed over 24 h in LD and 48 h in LL. In free running, samples were collected starting at ZT16. (b) effective absorption cross-section of PSII (σPSII), (c) maximum of the relative electron transfer rate (rETR max), (d) initial light limited slope of rETR (alpha) and (e) maximal NPQ measured (NPQm). Dots, squares, triangles and diamonds represents the individual measures for biological replicates A to D, colored lines represent the moving average of all replicates (window of 4 h, n=4). White and gray regions represent light and dark periods, gray dashed regions represent subjective nights in free-run conditions.

Fig. S10

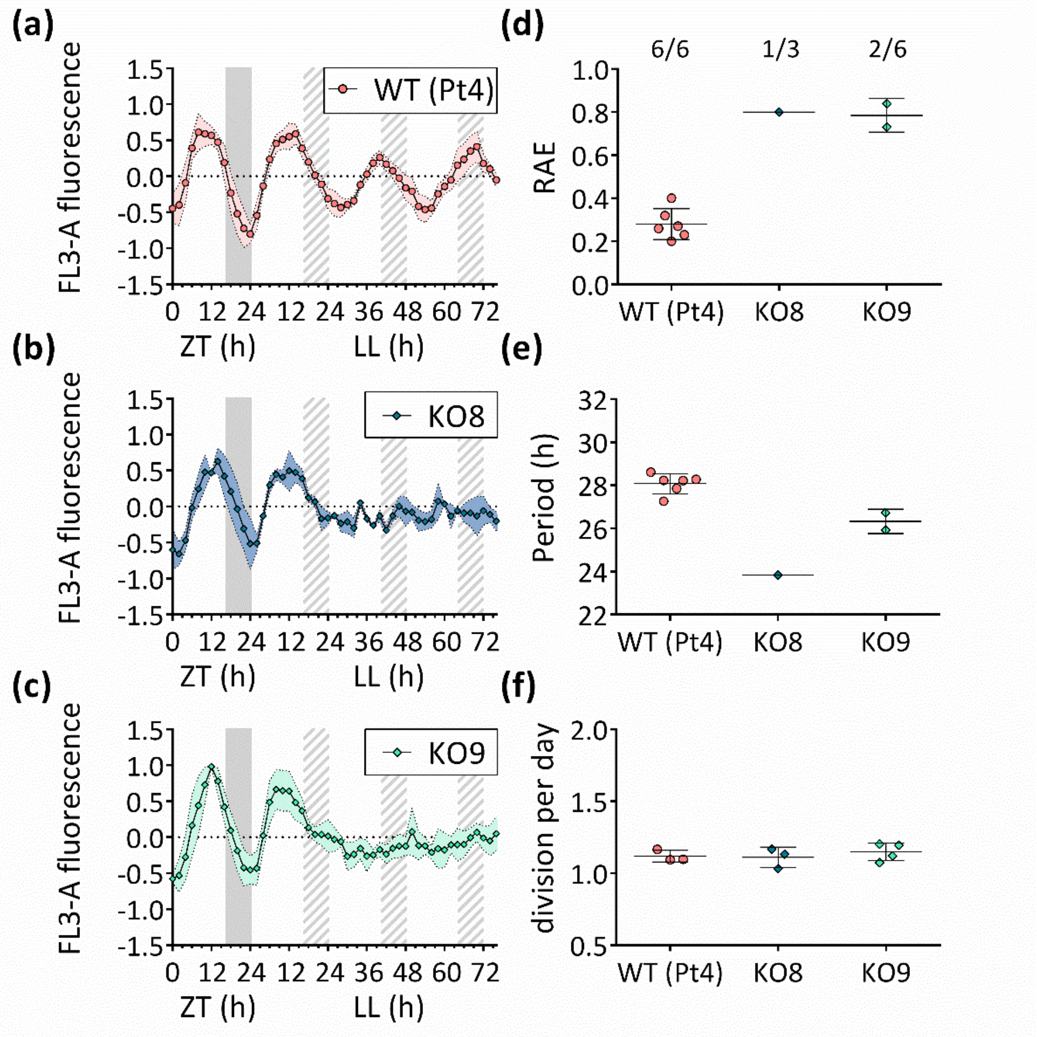

Fig. S10: Characterization of cellular fluorescence rhythmicity in *P. tricornutum* wild-type (WT) (Pt4) and the *Aureochrome1a* knock-out (KO8 and KO9) lines under 16 h : 8 h LD cycles, 25 µmol photons m^{-2} s^{-1} entrainment and following a switch to LL of 17 µmol photons m^{-2} s^{-1} for 3 days.

Normalised and baseline detrended circadian cellular FL3-A fluorescence profiles in WT (a), *Aureo1a* KO8 (b) and KO9 (c) (n>=6). Dots represent mean FL3-A fluorescence values, coloured envelopes represent Standard Deviation, SD. Gray dashed regions represent subjective nights in free-run conditions. (d) Relative amplitude of Error (RAE) of the FFT-NLLS (Fast Fourier Transform Non-Linear Least Square Algorithm) method fit for the lines found rhythmic in LL with the Enright Periodogram (EPR) algorithm test. The fraction of replicates that passes EPR test for all strains is noted above. (e) Period estimation obtained with FFT-NLLS method. (f) Division rate along the experiment (n>=6). Solid line indicates the mean and error bars the SD.

Fig. S11

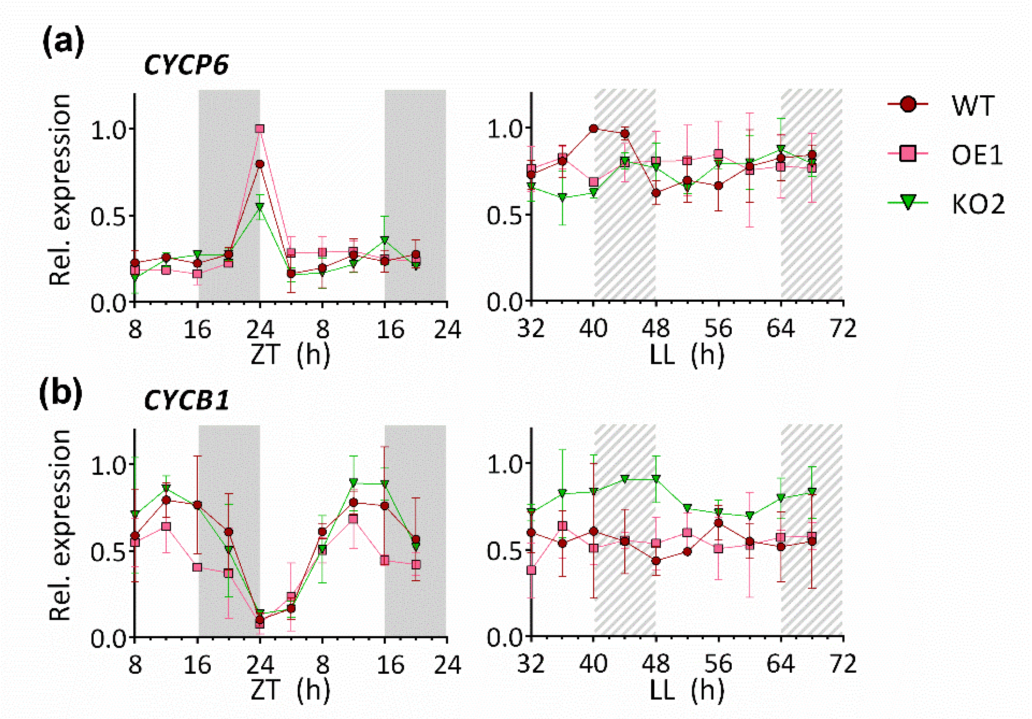

Fig. S11: Analysis of the rhythmic expression of selected genes by qRT-PCR in *P. tricornutum* wild-type (WT), the *RITMO1* overexpression (OE1) and knock-out (KO2) lines under LD cycles and LL conditions.

Expression profiles of the cyclin (a) *CYCP6* and (b) *CYCB1* for WT, KO2 and OE1 lines, normalized using the *RPS*, *TBP* reference genes. Expression values represent the average of three technical replicates for two biological replicates. Dots represent mean values, error bars represent Standard Deviation. Expression values are given relative to the maximum expression, where '1' represents the highest expression value of the time series. White and grey regions represent light and dark periods, grey dashed regions represent subjective nights in free-run conditions.

Fig. S12

CATTACTCGCATCCATTCTCAGGCTGTCTCGTCTCGTCTCCGaggtctcaggagaatgaggagGATGCCAATCTCGCCCATTCATGGTGTATACAAGTTCAAC
ATCCAAAGCTGGAACTTTTGGAAACAGAAAGAATGTCCGAATAGGGCACGGCGTGCCGTATTGTTGGAGTGGACTAGCAGAAAGTGAGGAAGG
CACAGGATGAGTTTCCTCGAGACACATAGCTTCAGCGTCGTGTAGGCTAGGCAGAGGTGAGTTTTCTCGAGACATACCTTCAGCGTCGTCTTCAC
TGTCACAGTCAACTGACAGTAATCGTTGATCCGGAGAGATTCAAAATTCAATCTGTTTGGACCTGGATAAGACACAAGAGCGACATCCTGACAT
GAACGCCGTAAACAGCAAATCCTGGTTGAACACGTATCCTTTTGGGGGCCTCCAGCTACGACGCTCGCCCCAGCTGGGGCTTCCTTACTATACAC
AGCGCATATTTCACGGTTGCCAGAAGTCAAGTCGAGGTCGATCCATATGACCACTCTTGACGACACGGCTTACCGGTACCGCACCAGTGTCCCGG
GGGACGCCGAGGCCATCGAGGCACTGGATGGGTCCTTCACCACCGACACCGTCTTCCGCGTCACCGCCACCGGGGACGGCTTCACCCTGCGGGA
GGTGCCGGTGGACCCGCCCCTGACCAAGGTGTTCCCCGACGACGAATCGGACGACGAATCGGACGACGGGGAGGACGGCGACCCGGACTCCCG
GACGTTCGTCGCGTACGGGGACGACGGCGACCTGGCGGGCTTCGTGGTCGTCTCGTACTCCGGCTGGAACCGCCGGCTGACCGTCGAGGACATC
GAGGTCGCCCCGGAGCACCGGGGGCACGGGGTCGGGCGCGCGTTGATGGGGCTCGCGACGGAGTTCGCCCGCGAGCGGGGCGCCGGGCACCTC
TGGCTGGAGGTCACCAACGTCAACGCACCGGCGATCCACGCGTACCGGCGGATGGGGTTCACCCTCTGCGGCCTGGACACCGCCCTGTACGACG
GCACCGCCTCGGACGGCGAGCAGGCGCTCTACATGAGCATGCCCTGCCCCTGAGCGGCCGACGGTATCGATAAGCTTGATATCGAATTCCTGCA
GCCCGGGGGATCCACTAGTTCTAGAGCGGCCGCAACAACTACCTCGACTTTGGCTGGGACACTTTCAGTGAGGACAAGAAGCTTCAGAAGCGTG
CTATCGAACTCAACCAGGGACGTGCGGCACAAATGGGCATCCTTGCTCTCATGGTGCACGAACAGTTGGGAGTCTCTATCCTTCcgctagcaaggagTC
GTTGTGAGCTGAAACTTCTCAAGAAGACAGTGAATCACTCGTGGGAACCTTGTATGTATTTGCTGTTGTATCGCCGCAGCAAGGTCTTGCCTGGG
AATACGCTGTCGAAAAGCTGTGCTTCGTGTTACAGCGATGACGACGAAAACGGCAATACTCCAAGAAACCGCTGGTACTCACACTGATTGCGCG
ACGGTGAGGTTGGTTGAAGGCGAAACCATACATGAAAAGCGCCACGTCGAAATTAAAAGGAAACTGCGACATCGTGGGCACTAGTCGAATTCA
GTTGAAAGACATTTTATTTAGTCGAAACACCAAAGAAGAAACGCAAATGACAGCTGCTCGAAACCATTACCTAGCCGAAAACTACAATCCCTCG
CTTGGCTTTTCACCACGTCACCTCCCTACTTATAGCCCTGCTTCTCCATGGTGAAGCGGAGTACCAGTTTTGATCTATGCGAATTTGTCTTTGAAG
GTCAGCTTCAGAAGGGATTCGGACGTGGTGCAACACGACTTTGATCCAGCCAATCAATCATGATAAACCAAGTCGTAGTTGCAGCAAACCAAAT
GGTAACACGCACGCGGATTGTGCTGGTGTGTTGACCCTACACATGTGGTACCCGTGGCTGCTACTCCTGGCCATGGAAGAGATATCGTTGCTGCT
GTTGCCCCAAAGCGATCGACACGTACTACTGTGTACTCAGTTCCCCGGTTCACCATTCCCTTTTAATTTTGCAGCTTTCTTGTCCGATCCCTTTCGC
TTTGAAGGGCTAGTTCGAATATTCGAATAGAGCCACCGGAAGTCGTAGCTGACCGAATACAATTAATGAATAAGCCAGGACAGCGGGGAACGTC
CCCGCAGTACAATGTGCGTGGGCTCTCCAGTGATCTCGCGACCATGAGTGATTTTCAGTTTTCGGCTTCCTTGGATGCTCTTTTGTACGGGGATAG
CATGACAGGCTCTGCTCCTAGTCAAGACAATCGCTACGGTTCGGAATCGTCCACGTCTCCAGCGAATAGCGAACCGCCTGCTTTCGCGAATCTTG
AGACGAATAGTAGTACGATGAGCACAAGCGCAAAAAGTAGTACGAGAAAGAGCTCTCATCACCATCAGCAAGGGAAGCGAAGTAGCTCCGGGA
TCATCACAGCCACTACTCTTGGCGTTGACGAGTCGGAGCAACCCCAGATAGTACAGAAGAAAACGCTGCCCTTGCGCTCAAGTGTAAATGCTGA
GTCTTCGCAAGTTGGGGCATCGTGCGTCGTTCTACCGCAACCATCCAAAATACCGGTAGATACCGCTCTGATGCCGAGTGGGGCTAGAAACGGA
GGATTTCCCGTACCGCAACTTTTCGGAAAAGTGGTGACTTTTGAAAATAGCCCACTTGCTTCAGCCACTAGTACTCATAGCAGCAGCGACAAGAC
TCCTACAACCAGCGGATCGAGCGAATCTGTCACCAGCGCTCGTATTTCAGGGAAACGAACATTCGAGCGGGGACAAGTGTCTGAAGACGAAAGC
GACCGCGAACGTCGTCGTTACGATCGCAACGCCCGCGAGCAGCAAAGGTCACAGCAAATCACTGTTCAGATAGACGTTCTGAAGAACGTTCTCA
CGGAAGCCAATGTCCAATTTAAGCCGGACAAATACAGTACATTGGTTACGGTGGTGGATTATGTCAAGACACTCCAAGAGCGATCGACTTTGCT
TGATCAAGAACACAAGAAGTTGTTGGACACCATTAGCAAGACAACAGAAATTGTCAATGGTTTGTACGTTCCCGCGGAGTCATCATCGTCTGAT
GTTTTATCTGGCCCATGTAGCCGTCCAGAACCGACACCCGAAGACGAAACTCTAGTTTTTGTCCGTGGTCTTGATTACAAGGCTGTTTTTGCTTTC
TGCCCGTTTGCCTGCGCCATCGCAAACATCGATGGTCGTTTTCTCGACTGTAATCAAGATTTTGAAAATATTACTGGCTACACTCGGGATGAGCTC
TTACCGCTTGAGGCTTCCTCTAGTGATCAGCATGCGTATTTGACACCCTTGCCCACTACAAGTGGAGACGTTATCGAGAGTACACAACCGCGGAA
TCTTTCCCTTTTCATTGTCTTGAATCAGGATCATATGCAGGACGTTTTCGGGGCCATGAGAGACATGTTGCGCCGTCCGTTTCCTGGAGAAATACG
GCACGATTCTTGGTCCCACCCGGTACATCTCAATCGTTTTCCCGATACTTTGGTAGGTTTTCCCTCGGCCTTAACGGTATGGCGATCTTCATAATG
ATTTTTCTCAAAAGTCTTGTCTTCCTTTAAACTTTACAGGTCAATATCAAAGTCAGTTTGGTACGAACAGCTCAGCATAGTCCTCGGTTTTTTAAT
GTGGCGTTGGTGCCATCAGACGATGCAGCGAAGCTGTCAGGTATGGTGAGCAAGGGCGAGGAGCTGTTCACCGGGGTGGTGCCCATCCTGGTCG
AGCTGGACGGCGACGTAAACGGCCACAAGTTCAGCGTGTCCGGCGAGGGCGAGGGCGATGCCACCTACGGCAAGCTGACCCTGAAGCTGATCT
GCACCACCGGCAAGCTGCCCGTGCCCTGGCCCACCCTCGTGACCACCCTGGGCTACGGCCTGCAGTGCTTCGCCCGCTACCCCGACCACATGAAG
CAGCACGACTTCTTCAAGTCCGCCATGCCCGAAGGCTACGTCCAGGAGCGCACCATCTTCTTCAAGGACGACGGCAACTACAAGACCCGCGCCG
AGGTGAAGTTCGAGGGCGACACCCTGGTGAACCGCATCGAGCTGAAGGGCATCGACTTCAAGGAGGACGGCAACATCCTGGGGCACAAGCTGG
AGTACAACTACAACAGCCACAACGTCTATATCACCGCCGACAAGCAGAAGAACGGCATCAAGGCCAACTTCAAGATCCGCCACAACATCGAGG
ACGGCGGCGTGCAGCTCGCCGACCACTACCAGCAGAACACCCCCATCGGCGACGGCCCCGTGCTGCTGCCCGACAACCACTACCTGAGCTACCA
GTCCGCCCTGAGCAAAGACCCCAACGAGAAGCGCGATCACATGGTCCTGCTGGAGTTCGTGACCGCCGCCGGGATCACTCTCGGCATGGACGAG
CTGTACAAGTGATAAGCTTGAGATGCAAATTATGTCTTTCGGCGACGGGTACACGTGTACATTACATATCACGATTGTGTGCTTAGAGAAAGTCC
CTTTGTAACCTCCTTTGCTCAATATTCAACCATCTCCTTGCATGCATTCGTTGCTGTCGTAAACCAGATTCAACACACGAGCTTTGACGTCAAGGC
TGCGCTTTTTACTAGCTAGTGTGATATCGCCTTACCTCGAAAGAAGGCAAACAATTCAAAAGTAACGCTTGCCCGGGGAGGGTTTGCGCTTCGAG
TACCAACGTGGCTGTACACTTCACAATTAGTGATTAGAGAAACACAGAGAAGTAGTAAATTGTGAATTCTTTGCTTATCATTACGAAAGCAGGC
AATAGTTATTTTCCATAGCGACGTTGCCTTTTAGCTGCGTATGACCGAATGATTTCCACCAAATCCGCTTTTTGTAGTTCAGGCCAGTTTTTCTCCA
AGAAAAAGAGCTCTGCGTAAGCGATCTGCCACAACAAAAAATTGCTCAATCGGAATTCTCCCGAAGTACGAATGACGACATcgctatacggagGGGAA
CAAAGGATTGTGCAGTGCCATACGACAAGTTCGCCTCAATCGCCAAACCCCTTGACTGTGCTTCCCGAGCGGAAACATTTAGATTGGCTACTACA
ACCGTTGCTCTCCGGGCGGATAAGAGAATAAATCTGGATTCGGCAGAGTAGTCGGCCTTATCATCAGTTAGATGTTGATAATCCCGTTGGGCAGG
TCACAcgctcagaggagGGGAACAAAGGATTGTGCAGTGCCATACGACAAGTTCGCCTCAATCGCCAAACCCCTTGACTGTGCTTCCCGAGCGGAAAC
ATTTAGATTGGCTACTACAACCGTTGCTCTCCGGGCGGATAAGAGAATAAATCTGGATTCGGCAGAGTAGTCGGCCTTATCATCAGTTAGATGTT
GATAATCCCGTTGGGCAGGTCACAcgctaggtatacttgagaccaCCCCAGGATACATAGATTACCACAACTCCGAGCCCTTCCACCcacagaatcaggggataacgcagg
aaagaacatgtgagcaaaaggccagcaaaaggccaggaaccgtaaaaaggccgcgttgctggcgtttttccataggctccgcccccctgacgagcatcacaaaaatcgacgctcaagtcagaggtggcgaaacccgacaggactataaa
gataccaggcgtttccccctggaagctccctcgtgcgctctcctgttccgaccctgccgcttaccggatacctgtccgcctttctcccttcgggaagcgtggcgctttctcatagctcacgctgtaggtatctcagttcggtgtaggtcgttcgctc
caagctgggctgtgtgcacgaaccccccgttcagcccgaccgctgcgccttatccggtaactatcgtcttgagtccaacccggtaagacacgacttatcgccactggcagcagccactggtaacaggattagcagagcgaggtatgtaggc
ggtgctacagagttcttgaagtggtggcctaactacggctacactagaaggacagtatttggtatctgcgctctgctgaagccagttaccttcggaaaaagagttggtagctcttgatccggcaaacaaaccaccgctggtagcggtggtttttt
gtttgcaagcagcagattacgcgcagaaaaaaaggatctcaagaagatcctttgatcttttctacggggtctgacgctcagtggaacgaaaactcacgttaagggattttggtcatgcattctaggtgattaTTATTTGCCGACTAC
CTTGGTGATCTCGCCTTTCACGTAGTGGACAAATTCTTCCAACTGATCTGCGCGCGAGGCCAAGCGATCTTCTTCTTGTCCAAGATAAGCCTGTCT
AGCTTCAAGTATGACGGGCTGATACTGGGCCGGCAGGCGCTCCATTGCCCAGTCGGCAGCGACATCCTTCGGCGCGATTTTGCCGGTTACTGCGC
TGTACCAAATGCGGGACAACGTAAGCACTACATTTCGCTCATCGCCAGCCCAGTCGGGCGGCGAGTTCCATAGCGTTAAGGTTTCATTTAGCGCC
TCAAATAGATCCTGTTCAGGAACCGGATCAAAGAGTTCCTCCGCCGCTGGACCTACCAAGGCAACGCTATGTTCTCTTGCTTTTGTCAGCAAGAT
AGCCAGATCAATGTCGATCGTGGCTGGCTCGAAGATACCAGCAAGAATGTCATTGCGCTGCCATTCTCCAAATTGCAGTTCGCGCTTAGCTGGAT
AACGCCACGGAATGATGTCGTCGTGCACAACAATGGTGACTTCTACAGCGCGGAGAATCTCACTCTCTCCAGGGGAAGCCGAAGTTTCCAAAAG
GTCGTTGATCAAAGCTCGCCGCGTTGTTTCATCAAGCCTTACGGTCACCGTAACCAGCAAATCAATATCACTGTGTGGCTTCAGGCCGCCATCCA
CTGCGGAGCCGTACAAATGTACGGCCAGCAACGTCGGTTCGAGATGGCGCTCGATGACGCCAACTACCTCTGATAGTTGAGTCGATACTTCGGC
GATCACCGCTTCCCTCATGCGAAACGATCCTCATCCTGTCTCTTGATCAGATATTGATCCCCTGCGCCATCAGATCCTTGGCGGCAAGAAAGCCA
TCCAGTTTACTTTGCAGGGCTTCCCAACCTTACCAGAGGGCGCCCCAGCTGGCAATTCCGGTTCGCTTGCTAAGCTTGCATGCCTGCAAGTCGAC
TCTAGAGGAgcggtgatcacaggcagcaacgctctgtcatcgttacaatcaacatgctaccctccgcgagatcatccgtgtttcaaacccggcagcttagttgccgttcttccgaatagcatcggtaacatgagcaaagtctgccgcct
tacaacggctctcccgctgacgccgtcccggactgatgggctgcctgtatcgagtggtgattttgtgccgagct

Fig. S12: Sequence of the pL2-1 plasmid used to transform *P. tricornutum RITMO1* knock-out KO2 to generate the complemented KO2-C2 strain (RITMO1p::RITMO1g:Venus::RITMO1t).

Fig. S13

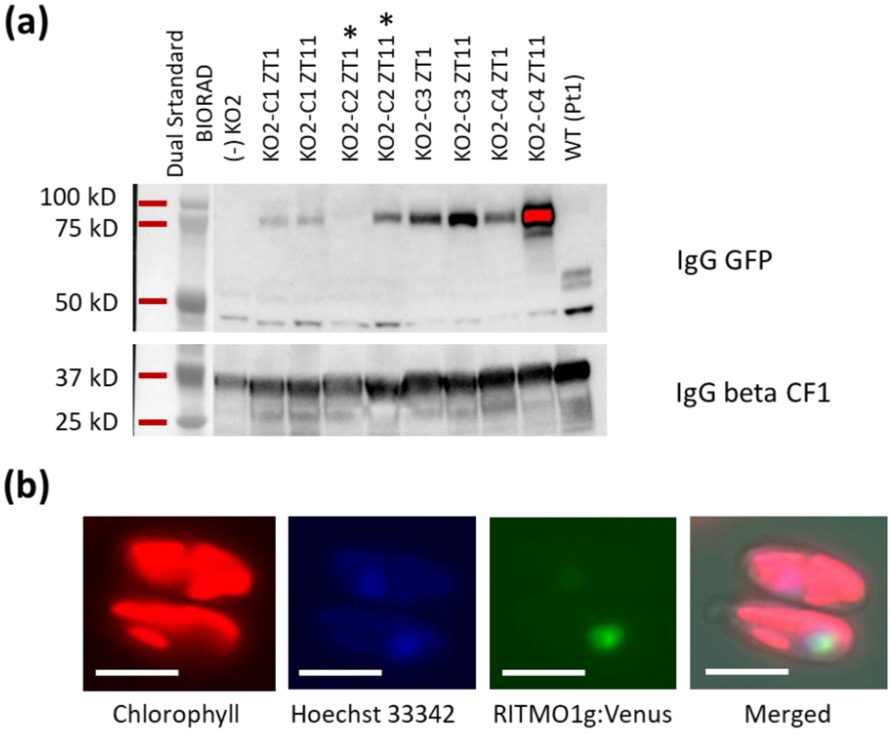

Fig. S13: Selection of complemented *RITMO1* lines in *RITMO1* KO2 mutant.

(a) Western blot analysis of transgenic lines of *RITMO1* KO2 mutant transformed with the RITMO1p::RITMO1g:Venus::RITMO1t construct by biolistic. In this experiment, cells were grown in 12h:12h light-dark (LD) cycles at 25 µmol photons m^{-2} s^{-1} and collected for the analysis 1h after light onset (ZT 1) and 1 h before the dark (ZT11). KO2 is the original mutant strain (negative control, -), KO2-C indicates the independent complemented lines. Top are the results obtained with the IgG anti-GFP (1/2000) and down with an IgG anti-beta CF1 (1/20000), used as a loading control. The KO2-C2 line used in Fig. 7 is indicated with an asterisk.
(b) Fluorescence microscopy of *P. tricornutum* cells expressing the RITMO1g:Venus protein under the control of the endogenous *RITMO1* promoter. Samples imaged on exponential phase cells grown in 16 h : 8 h LD 25 µmol photons m^{-2} s^{-1} at ZT15. (Scale bar: 5 µm). For chlorophyll autofluorescence and Venus fluorescence cells were excited at 510 nm and detected at 650–741 nm and 529–562 nm, respectively. Nuclear DNA stained with Hoechst 33342 were visualized by illumination at 405 nm and detection at 424–462 nm.

Fig. S14

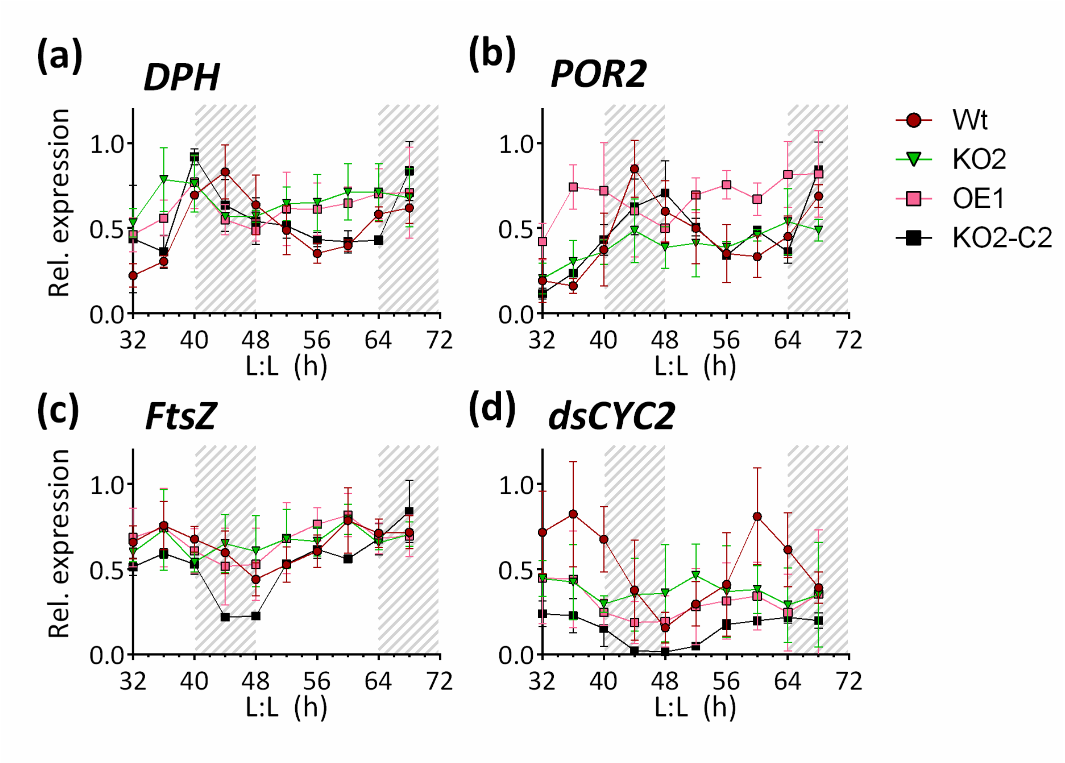

Fig. S14: Analysis of the rhythmic expression of selected genes by qRT-PCR in *P. tricornutum* wild-type (WT), *RITMO1* ectopic overexpression (OE1), *RITMO1* knock-out KO2 and the KO2-C2 complemented strains (n>=6) in free running conditions.

Expression profiles of the photoreceptors *DPH* (a) and *CPF1* (b), the *FtsZ* (c) and the cyclin ds*CYC2* (d) for WT, KO2, OE1 and KO2-C2 lines. Expression values represent the average of three biological triplicates ±Standard Deviation (SD), normalized using the *RPS* and *TBP* reference genes. Expression values are given relative to the maximum expression for each gene, where '1' represents the highest expression value of the time series. Dots represent mean values, error bars represent SD (n=3). Grey dashed regions represent subjective nights in free-run conditions.

Fig. S15

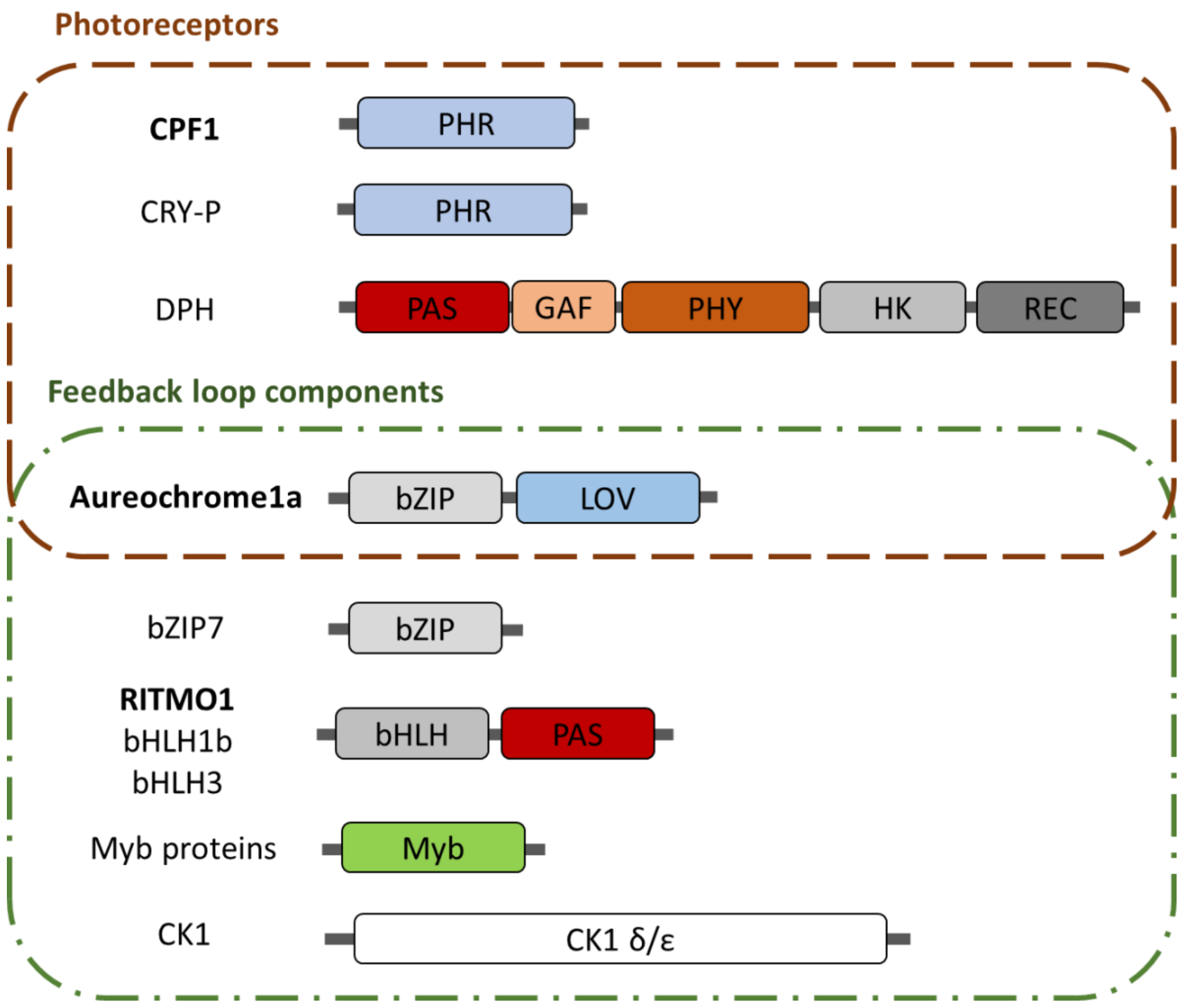

Fig. S15: Scheme of the validated and putative components of the diatom circadian clock system.

The figure includes diatom photoreceptors, putative components of the input pathway, and clock regulatory loop factors identified in this study, and from diatom genomic studies: Coesel *et al.*, 2009; Annunziata *et al.*, 2019; Madhuri *et al.*, 2024. Validated clock components are shown in bold. The involvement of CPF1 in the circadian clock has so far only been shown in heterologous mammalian cell system (Coesel *et al.*, 2009). From top to bottom: proteins with photoreceptor domains CPF1 (Phatr3_J27429), Cry-P (Phatr3_J54342), DPH (Phatr3_J54330), the Aureochrome1a with both bZIP transcription factor (TF) and LOV photoreceptor domains (Phatr3_J8113), TFs containing bZIP domain bZIP7 (Phatr3_J48800), the animal-like bHLH-PAS domains in RITMO1 (Phatr3_J44962), bHLH1b (Phatr3_J44963, and the bHLH domain protein bHLH3 (Phatr3_J42586); untested genes containing SANT/Myb domain, such as plant clock components. Diatom genomes also contain several proteins of the plant clock system with a CCT domain. However, these proteins lack other domains necessary for circadian clock function and are therefore not included in the proposed scheme. CK1 δ/ε type genes, known to be cross-taxon post-translational regulators of the circadian clock, have also been found (Phatr3_J42322, Phatr3_J51110). The Phatr3 identifiers of the different components in the *P. tricornutum* genome are given in this legend (https://www.diatomicsbase.bio.ens.psl.eu/) (Villar *et al.*, 2025).

Bibliography

Agier N, Fischer G. **2016**. A versatile procedure to generate genome-wide spatiotemporal program of replication in yeast species. In: Devaux F, ed. Methods in Molecular Biology. Yeast Functional Genomics: Methods and Protocols. New York, NY: Springer, 247–264.
Annunziata R, Ritter A, Fortunato AE, Manzotti A, Cheminant-Navarro S, Agier N, Huysman MJJ, Winge P, Bones AM, Bouget F-Y, *et al.* 2019. bHLH-PAS protein RITMO1 regulates diel biological rhythms in the marine diatom Phaeodactylum tricornutum. *Proceedings of the National Academy of Sciences* **116**: 13137–13142.
Blommaert L, Chafai L, Bailleul B. **2021**. The fine-tuning of NPQ in diatoms relies on the regulation of both xanthophyll cycle enzymes. *Scientific Reports* **11**: 12750.
Coesel S, Mangogna M, Ishikawa T, Heijde M, Rogato A, Finazzi G, Todo T, Bowler C, Falciatore A. **2009**. Diatom PtCPF1 is a new cryptochrome/photolyase family member with DNA repair and transcription regulation activity. *EMBO Reports* **10**: 655–661.
Falciatore A, Casotti R, Leblanc C, Abrescia C, Bowler C. **1999**. Transformation of Nonselectable Reporter Genes in Marine Diatoms. *Marine Biotechnology* **1**: 239–251.
Gorbunov M, Shirsin E, Nikonova E, Fadeev V, Falkowski P. **2020**. A multi-spectral fluorescence induction and relaxation (FIRe) technique for physiological and taxonomic analysis of phytoplankton communities. *Marine Ecology Progress Series* **644**: 1–13.
Madhuri S, Lepetit B, Fürst AH, Kroth PG. **2024**. A knockout of the photoreceptor PtAUREO1a results in altered diel expression of diatom clock components. *Plants* **13**: 1465.
Pollak B, Matute T, Nuñez I, Cerda A, Lopez C, Vargas V, Kan A, Bielinski V, von Dassow P, Dupont CL, *et al.* 2020. Universal loop assembly: open, efficient and cross-kingdom DNA fabrication. *Synthetic Biology* **5**: ysaa001.
Serôdio J, Lavaud J. **2011**. A model for describing the light response of the nonphotochemical quenching of chlorophyll fluorescence. *Photosynthesis Research* **108**: 61–76.
Villar E, Zweig N, Vincens P, Carvalho HC de, Duchene C, Liu S, Monteil R, Dorrell RG, Fabris M, Vandepoele K, *et al.* 2025. DiatOmicBase, a gene-centered platform to mine functional omics data across diatom genomes. *Plant Journal:* **121**(6):e70061.
